# ChatGPT Clinical Use in Mental Health Care: Scoping Review of Empirical Evidence

**DOI:** 10.2196/81204

**Published:** 2025-12-24

**Authors:** Raluca Balan, Thomas P Gumpel

**Affiliations:** 1 Seymour Fox School of Education, Hebrew University of Jerusalem, Mount Scopus, Jerusalem, 94554 11, Israel, 972 0545443079

**Keywords:** artificial intelligence, ChatGPT, clinical decision making, counseling, diagnostic, evaluation, mental health, prognosis, Preferred Reporting Items for Systematic Reviews and Meta-Analyses, PRISMA

## Abstract

**Background:**

As mental health challenges continue to rise globally, there is an increasing interest in the use of GPT models, such as ChatGPT, in mental health care. A few months after its release, tens of thousands of users interacted with GPT-based therapy bots, with mental health support identified as the primary use case. ChatGPT offers scalable and immediate support through natural language processing capabilities, but their clinical applicability, safety, and effectiveness remain underexplored.

**Objective:**

This scoping review aims to provide a comprehensive overview of the main clinical applications of ChatGPT in mental health care, along with the existing empirical evidence for its performance.

**Methods:**

A systematic search was conducted in 8 electronic databases in April 2025 to identify primary studies. Eligible studies included primary research, reporting on the evaluation of a ChatGPT clinical application implemented for a mental health care–specific purpose.

**Results:**

In total, 60 studies were included in this scoping review. The results highlighted that most applications used generic ChatGPT and focused on the detection of mental health problems and counseling and treatment. At the same time, only a minority of studies investigated ChatGPT use in clinical decision facilitation and prognosis tasks. Most of the studies were prompt experiments, in which standardized text inputs—designed to mimic clinical scenarios, patient descriptions, or practitioner queries—are submitted to ChatGPT to evaluate its performance in mental health-related tasks. In terms of performance, ChatGPT shows good accuracy in binary diagnostic classification and differential diagnosis, simulating therapeutic conversation, providing psychoeducation, and conducting specific therapeutic strategies. However, ChatGPT has significant limitations, particularly with more complex clinical presentations and its overly pessimistic prognostic outputs. Nevertheless, overall, when compared to mental health experts or other artificial intelligence models, ChatGPT approximates or surpasses their performance in conducting various clinical tasks. Finally, custom ChatGPT use was associated with better performance, especially in counseling and treatment tasks.

**Conclusions:**

While ChatGPT offers promising capabilities for mental health screening, psychoeducation, and structured therapeutic interactions, its current limitations highlight the need for caution in clinical adoption. These limitations also underscore the need for rigorous evaluation frameworks, model refinement, and safety protocols before broader clinical integration. Moreover, the variability in performance across versions, tasks, and diagnostic categories also invites a more nuanced reflection on the conditions under which ChatGPT can be safely and effectively integrated into mental health settings.

## Introduction

Mental health problems affect 1 in 2 people globally, leading to significant impairments in daily functioning and well-being [1]. By 2030, the economic burden is expected to reach US $6 trillion, surpassing that of cancer, diabetes, and respiratory diseases combined [2]. Despite efforts to improve services, barriers like provider shortages, waitlists, geographic access, and stigma persist, leaving many without adequate care [3]. Artificial intelligence (AI) is increasingly recognized as an alternative revolutionary technology in mental health care that has the potential to surpass these significant gaps [4]. Among AI technologies, one of the most recent significant developments is ChatGPT, a conversational system based on the large language model (LLM) GPT, developed by OpenAI, that processes and analyzes large amounts of data to generate responses to user inquiries. ChatGPT can mimic human-like dialogues and perform complex functions, making it a suitable tool for assisting various mental health care tasks. Moreover, its ability to provide immediate, anonymous, and scalable support is particularly beneficial in addressing gaps in mental health services, especially in regions with limited access to professional care [5,6].

Importantly, ChatGPT builds on earlier digital mental health platforms such as Woebot, Wysa, and Tess, which demonstrated feasibility and efficacy in providing psychoeducation, stress management, and mood support through scripted dialogues [7-9]. While these tools proved effective for specific tasks, their reliance on predefined responses limited flexibility and adaptability. ChatGPT represents the next step in this evolution, enabling more naturalistic conversations and broader applications, while also introducing new challenges.

Since its release, a growing body of research has focused on developing and testing various applications of ChatGPT in mental health care. ChatGPT capabilities include identifying mental health problems [10-12], determining the severity of the problems [13], assisting mental health practitioners in assessing the course of the treatment [14], prognostic [15], performing case conceptualization [16], or cognitive behavioral therapy (CBT) techniques such as cognitive restructuring [17]. Even more outstanding applications of ChatGPT in mental health consist of its use as a therapy enhancement for Attention Deficit Hyperactivity Disorder (ADHD) treatment [18] or even as a standalone psychotherapist for the clinical populations presenting with anxiety disorders [19].

Besides the tremendous benefits, there is also a lot of skepticism surrounding the use of ChatGPT as a tool for enhancing mental health care. Some authors note data privacy violations, the tendency to present confidently false information, or the underestimation of the risk of suicide attempts as central issues in integrating ChatGPT into mental health care [20,21]. Additionally, other researchers question the ability of the last iterations of GPT to display empathy and to recognize emotional reactions. These skills are crucial in conducting clinical assessments or in providing psychological interventions [22]. Therefore, the trend of using ChatGPT without sufficient attention to its limitations and risks can be detrimental, given the growing public awareness and easy access to ChatGPT [23].

Several reviews addressing the role of generative AI and LLMs in psychiatry and mental health care have been published to date, showing that although there are clear benefits, generative AI is not yet ready for standalone use in the field [21,24,25]. While numerous AI tools hold potential value for clinical practice, ChatGPT has emerged as the most prominent LLM in the health care domain, surpassing alternatives such as Google’s Gemini [26]. As of January 2024, the ChatGPT Store reported tens of thousands of interactions involving GPT-based therapy bots, with 1 in every 25 users seeking mental health support as a primary use case [27,28].

Notably, only 1 review has specifically examined ChatGPT within the context of psychiatry [29]; however, this review does not comprehensively capture empirical evidence on its clinical applications. Given the rapid evolution of ChatGPT models, which increasingly feature enhanced capabilities and novel interaction modalities, even reviews conducted within the past year may already be outdated, omitting key advancements that could substantially affect performance in mental health practice. Considering the significant benefits and the potential risks associated with integrating ChatGPT into mental health care, a comprehensive and up-to-date synthesis of the evidence is warranted.

Therefore, our aim is to conduct a scoping review exploring the main clinical applications of ChatGPT in mental health care and its current empirical evidence. More specifically, this review is guided by 2 research questions: (1) What are the characteristics of the clinical applications of ChatGPT in mental health care? (2) What is the current empirical evidence regarding the clinical applications of ChatGPT in mental health care?

The findings of this review can inform various stakeholders, including researchers, clinicians, and support seekers, about the potential uses, implications, and efficacy of ChatGPT technology in the field of mental health.

## Methods

### Data Charting and Categorization

The scoping review was conducted in line with the PRISMA (Preferred Reporting Items for Systematic Reviews and Meta-Analyses) guidelines for conducting systematic scoping reviews (Checklist 1) [30]. The protocol for this scoping review was prospectively registered in Open Science Framework [31].

### Eligibility Criteria

We included primary research that evaluated a ChatGPT application, implemented for a mental health care–specific purpose, and reported on a performance-related outcome. Performance-related outcomes were operationalized as any qualitative or quantitative data regarding, but not limited to, accuracy, precision, acceptability, feasibility, safety, usability, efficacy, strengths, or limitations of ChatGPT performing a specific task in the mental health care landscape. We focused only on clinical applications of ChatGPT, such as prediction, detection of mental health problems, psychological interventions, or clinical decision-making, while excluding studies investigating the use of ChatGPT for research, educational, technical, or administrative purposes. Reviews, as well as studies that describe the development of a ChatGPT application without reporting any performance-related outcomes, were excluded. Studies focusing solely on other generative AI technologies (eg, Claude, Copilot, and Gemini) were also excluded.

### Search Strategy

The first author conducted a search in April 2025 in multidisciplinary and specific domain databases (Web of Science, PubMed, Scopus, PsycINFO, Association for Computing Machinery Digital Library, IEEE Xplore, Open Access Theses and Dissertations, EBSCO, and ProQuest). A sample of the search strategy used is presented in Multimedia Appendix 1.

### Study Selection

Screening of articles for inclusion was performed in 2 stages: title and abstract review and full article review, conducted independently by 2 reviewers. Following an initial screening of titles and abstracts, full texts were obtained and screened by 2 reviewers. Any divergences were solved through discussions between the 2 reviewers. The screening procedure was piloted under Cochrane guidelines, with a random sample of studies for both abstract and full text [32].

### Data Items and Charting

A standardized data extraction form was designed before data charting. The form was piloted and refined with the screening team. Similar to the study selection process, the 2 reviewers independently conducted the process of data extraction, with discrepancies being resolved by discussions and consensus.

From the included studies, relevant information was charted in an Excel (Microsoft) spreadsheet: (1) type of publication (peer-reviewed article, conference proceedings, working papers, etc), (2) purpose of application (detection/assessment, therapeutic application, decision making, and prognosis), (3) mental health problem focus, (4) age category of intended end users, (5) type of ChatGPT model (standard, custom instruction, custom GPT), (6) study design/methodology (prompt study, quasi-experimental, controlled study, study case, (7) participants, (8) comparison element (MH practitioners, other AI models), (9) outcomes assessed, and (10) the main findings. A detailed overview of the definitions for each item, along with its corresponding categories, is provided in Table S1 of the Multimedia Appendix 2.

### Synthesis of the Results

Consistent with methodology for scoping review, data were synthesized using a descriptive and thematic approach [33]. We first conducted a numerical summary of study characteristics (eg, publication type, mental health focus, study design, ChatGPT version, etc). Then, we grouped findings by major application domains (detection, counseling/treatment, clinical decision support, and prognosis) following a deductive approach, where each study was assigned to the predetermined categories developed during the protocol stage. Finally, we presented a narrative synthesis of main findings to identify overarching patterns in performance, comparisons with mental health professionals or other AI systems, as well as variations across tasks and model versions, and evidence gaps. Regarding the relative performance of ChatGPT compared to mental health experts or other AI models, this reflects the comparative conclusions reported in individual studies, rather than a statistical synthesis across studies.

## Results

### Study Search

**Figure 1.**
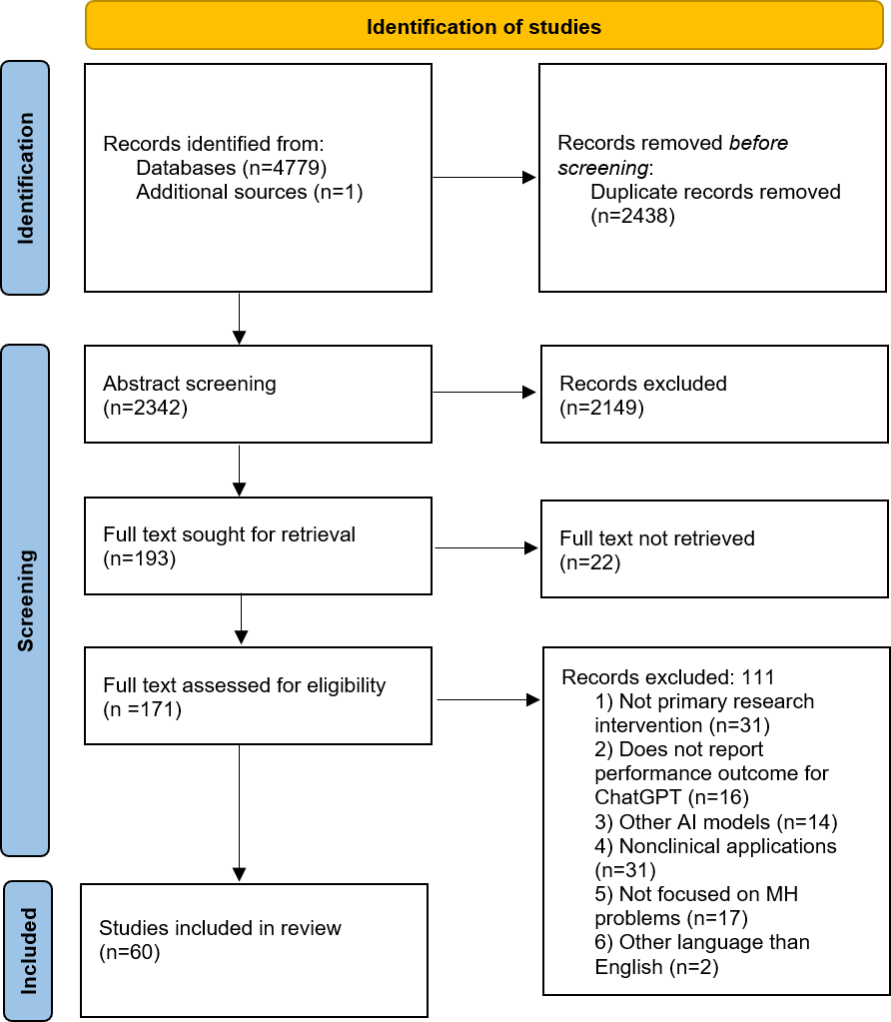
PRISMA (Preferred Reporting Items for Systematic Reviews and Meta-Analyses) flowchart.

The detailed study selection process is presented in Figure 1, the PRISMA flowchart. A total of 4780 articles were identified in the search. After eliminating duplicates, 2342 abstracts were screened for title and abstract, with an additional 2149 articles being excluded. Out of the 193 remaining articles, 172 full-text copies were retrieved that were screened in full. This resulted in 60 articles being included in the current review. The detailed characteristics of the included studies are presented in Multimedia Appendix 3.

### Characteristics of Clinical Applications and Research

Summative results, as per characteristics of ChatGPT clinical applications and research, are detailed in Table 1. Most of the articles were published in peer-reviewed journals (n=47) [10-12,14-19,34-71], followed by conference proceedings (n=9) [13,36,72-78], and preprints (n=4) [79-82]. Regarding the purpose of the application, ChatGPT was predominantly employed as a tool for counseling and interventions in mental health care (n=29) [16-19,34-36,41,43,44,46,47,49,54-62,66-68,71,72,76,80], and detection/assessment of mental health problems (n=24) [10-13,15,15,42,48,51-53,63-65,69,70,73-75,77-79,81,82]. A few studies explored its application in supporting clinical decision-making (n=8) [14,15,37-39,42,50,52], respectively in prognosis (n=3) [15,40,83]. While a substantial portion of the studies addressed mental health in general (n=16) [11,16,17,34,35,37,38,43,53,55,58,59,71,72,76,80], others focused on specific conditions, including depression (n=15) [13,15,40,41,47,50,62,63,68,73,75,78,79,81,82], suicidality (n=12) [12,15,47,51,56,64,65,67,70,74,77,81], anxiety (n=8) [15,34,41,49,52,54,78,79], schizophrenia (n=4) [15,42,45,83], substance use disorders (n=3) [44,61,66], and autism spectrum disorders (n=3) [46,57,69]. Additionally, attention deficit hyperactivity disorder [18,36], and post-traumatic stress disorder [10,15] were each the primary focus of two studies (n=2), whereas individual studies addressed bipolar disorder [60], obsessive-compulsive disorder [48], insomnia [39], and self-harm [14].

**Table 1.** Summative results per characteristics of ChatGPT applications and research.

Category	Number of studies	Percentage (%)	Studies
Publication type	
Peer-reviewed journal	47	76	[10-12,14-19,34-71]
Conference proceedings	9	16	[13,36,72-78]
Preprints	4	6	[79-82]
Application purpose
Detection/assessment	24	40	[10-13,15,15,42,48,51-53,63-65,69,70,73-75,77-79,81,82]
Counseling and intervention	29	48	[16-19,34-36,41,43,44,46,47,49,54-62,66-68,71,72,76,80]
Clinical decision facilitation	8	13	[14,15,37-39,42,50,52]
Prognosis	3	5	[15,40,83]
Mental health focus			
General MH^a^	16	26	[11,16,17,34,35,37,38,43,53,55,58,59,71,72,76,80]
Depression	15	25	[13,15,40,41,47,50,62,63,68,73,75,78,79,81,82]
Anxiety	8	13	[15,34,41,49,52,54,78,79]
Suicide	12	20	[12,15,47,51,56,64,65,67,70,74,77,81]
Schizophrenia	4	6	[15,42,45,83]
Substance use disorders	3	5	[44,61,66]
ASD^b^	3	5	[46,57,69]
ADHD^c^	2	3	[18,36]
PTSD^d^	2	3	[10,15]
Bipolar disorder	1	1	[60]
OCD^e^	1	1	[48]
Insomnia	1	1	[39]
Self-harm	1	1	[14]
Age category end users			
Adults	56	93	[10-12,14-19,34-70]
Children and adolescents	4	6	[11,36,52,69]
ChatGPT type			
Standard	50	83	[11-16,19,34,37-48,50-53,55-70,72,73,76-83]
Custom instruction	4	6	[10,63,74,75]
Customized GPT	6	10	[18,36,47,49,54,71]
Study design			
Prompt experiments	50	83	[10-18,36-48,50-53,55-57,59-67,69,70,72-83]
Controlled trials	3	5	[49,58,68]
Uncontrolled trials	5	8	[19,34,35,54,71]
Study case	2	3	[42,43]
Direct involvement of participants			
General population	4	6	[43,49,68,71]
Clinical population	6	10	[19,34,35,42,54,58]
Comparison element			
MH experts	19	31	[11-13,15,35,38,40,45,46,50-52,56,58,59,66,68,69,83]
AI tools	21	35	[13,15,17,36,39,40,44,46,48,49,52,53,56,62,67,74,76,77,79,82,83]

a MH: Mental health

b ASD: autism spectrum disorder

c ADHD: attention deficit hyperactivity disorder

d PTSD: post-traumatic stress disorder

e OCD: obsessive compulsive disorder

The age category of the intended end users of the clinical applications consisted mostly of adults (n=56) [10-12,14-19,34-70], with only 4 studies evaluating the use of ChatGPT for detection, counseling, and clinical decision facilitation for mental health problems among children and adolescents [11,36,52,69]. Regarding the ChatGPT model specifications, most studies employed standard ChatGPT (n=50) [11-16,19,34,37-48,50-53,55-70,72,73,76-83]. Customized ChatGPTs were used in 6 studies [18,36,47,49,54,71], while 4 studies employed a custom instruction GPT model [10,63,74,75].

Most studies were designed as prompt experiments (n=50), in which the accuracy or the quality of ChatGPT-generated responses to various queries were evaluated, without involvement of human participants [10-18,36-48,50-53,55-57,59-67,69,70,72-83]. The designs of the remaining studies included uncontrolled clinical trials (n=5) [19,34,35,54,71], controlled trials (n=3) [49,58,68], and case reports (n=2) [42,43]. Only 10 studies enlisted participants to use/test ChatGPT as a part of an experimental setup. Among these 10 studies, adults from the general population were involved in 4 studies [43,49,68,71], while 6 other studies had participants from the clinical population [19,34,35,42,54,58]. The number of participants varied between 1 and 399. Participants were predominantly young adults with a high educational level. Dropout rates were generally low, except for 1 study that involved the elderly [68]. The performance in the specific clinical tasks conducted by ChatGPT was assessed by comparison with mental health practitioners (n=19) [11-13,15,35,38,40,45,46,50-52,56,58,59,66,68,69,83] or with other AI models (n=21) [13,15,17,36,39,40,44,46,48,49,52,53,56,62,67,74,76,77,79,82,83].

### Main Findings

The main findings for ChatGPT use in detection, counseling and intervention, clinical decision facilitation, and prognosis of mental health care are presented in Multimedia Appendix 4.

#### Detection

The performance in the detection of mental health problems was assessed in 24 studies. Outcomes included agreements rate between ChatGPT and mental health experts and accuracy in diagnosis, as expressed by the *F*_1_ metric, and defined as the harmonic mean of precision (the proportion of cases the model correctly identifies as positive out of all it labels as positive) and recall (the proportion of true positive cases correctly identified out of all actual positives) [84]. Most studies reported moderate to high accuracy in categorical decisions, such as determining whether an individual met criteria for a disorder detection and differential diagnosis between 2 disorders (anxiety versus depression, Asperger syndrome versus autism disorder), with *F*_1_ scores ranging between 0.5 and 0.9. However, low diagnostic accuracy (*F*_1_ scores below 0.5) was reported for more complex detection tasks consisting of estimating mental health problems’ severity (especially suicide risk) or assigning a psychiatric diagnosis in a very heterogeneous data set presentation [12,15,81].

When compared to mental health professionals, ChatGPT underperformed in 2 studies, underestimating the severity of depression, risk of suicide ideation, and attempts [12,13]. In contrast, 4 studies reported comparable or superior diagnostic accuracy in identifying schizophrenia, childhood anxiety, differentiating neurodevelopmental disorders, and mental health conditions from physical health problems [11,45,52,69].

Against other AI systems, ChatGPT showed comparable or superior accuracy in 6 studies, particularly for obsessive-compulsive disorder, anxiety, depression, and gender bias in depression [48,53,73,77,79,82]. However, 3 studies reported lower accuracy, especially in severity estimation, suicidality assessment, and recognition of childhood anxiety [13,52,74].

When considering the model versions, GPT-4 generally performs best, reaching good accuracy in several conditions such as depression, post-traumatic stress disorder, social phobia, and suicidal ideation, and showing strong sensitivity to clinical risk factors [15,48,65]. Still, it underperforms in some cases, like schizophrenia (*F*_1_=0.55) [15]. GPT-3.5 shows mixed results: it sometimes outperforms GPT-4 (eg, depression detection) [73], but often performs poorly without fine-tuning [63], and can fail severely in tasks such as suicidal ideation detection [15]. GPT-3.5 Turbo improves on standard 3.5 for depression (*F*_1_=0.86) but is weak in suicidality detection [81]. Cultural sensitivity differed between GPT-3.5 and 4, with GPT-3.5 integrated cross-cultural distinctions across all dimensions of suicide risk, whereas GPT-4 was sensitive only to the likelihood and fatality of attempts [70]. Overall, GPT-4 is the strongest model, while standard GPT-3.5 is the least reliable. Of all 3 studies, 3 also examined differences between standard and fine-tuned versions of GPT, with results consistently favoring fine-tuned models for mental health detection tasks [10,63,75].

#### Counseling and Intervention

The use of ChatGPT in psychological counseling and intervention was assessed in 29 studies. Most studies focused on the quality of the responses to counseling and intervention-related queries (n=13). Mixed results regarding quality of responses were reported in 7 studies [41,46,56,60,61,67], positive in 3 [55,59,62], and negative in other 3 studies [44,47,66]. Therapeutic abilities were rated high across 3 studies [18,36,80], low in 1 study [72], and mixed in another study [19]. More specifically, ChatGPT demonstrated moderate to high empathy, positive atmosphere, encouragement of autonomy, listening abilities, as well as flexibility in conversation [18,19,36,80]. The most frequent negative aspects were related to ethics and confidentiality concerns and limited referrals to external sources or evidence-based content [18,19,34,57,60,61,67].

Performance in conducting specific therapeutic tasks was evaluated in 3 studies. ChatGPT demonstrated potential to generate psychodynamic conceptualizations [16], while the evidence regarding its proficiency to conduct cognitive restructuring is mixed [17,76].

Only 4 studies investigated the efficacy of ChatGPT in reducing mental health problems [49,54,58,68]. Out of the 4 studies, 2 showed superior efficacy compared to the control group in reducing anxiety, while increasing self-compassion [49], and quality of life, respectively [58]. One study indicated no significant difference between ChatGPT and control in reducing tension [68]. Another uncontrolled study showed a significant pre- and post-reduction in anxiety for a customized version of ChatGPT [54].

When benchmarked to mental health experts, ChatGPT has a comparable or better performance in 4 studies, in terms of efficacy in symptom improvement, appropriateness of information, depth, and empathy [56,58,59,68]. ChatGPT exhibited lower performance than mental health experts in 3 studies, in terms of mental health-related information precision, usefulness, and relevance [35,46,66]. In comparison to other AI-powered tools, ChatGPT had a similar or superior performance in tasks related to counseling and intervention than Gemini, BARD, Google, Claude, and a rule-based chatbot specifically designed for mental health support [17,36,46,62], but underperformed Claude, Bing Copilot, and a specific AI-powered therapy role-play platform in 3 other studies [56,67,76].

GPT-4 generally shows the strongest performance, offering clinically relevant, empathetic, and evidence-aligned responses across various contexts, such as autism information, postpartum depression, substance use, and autism spectrum disorder support [46,61,62]. GPT-3.5 delivers mixed results, sometimes empathetic and safe [67], but prone to unsafe delays in referrals or limited therapeutic depth [47]. GPT-3 shows the weakest results overall, with limited impact beyond basic relaxation benefits compared to traditional therapies [68].

Of all 4 studies focused on the use of customized ChatGPTs, demonstrating high capabilities in queries related to general mental health and ADHD [18,36,71], but significant limitations in dealing with suicidal ideation [47].

#### Clinical Decision Facilitation

The use of ChatGPT in supporting clinical decision-making was examined across 8 studies. Most investigations assessed the alignment of ChatGPT’s treatment recommendations with evidence-based practices. Findings indicated that ChatGPT could generate clinically appropriate recommendations consistent with established guidelines for specific mental health conditions [14,37-39,42,50]. However, for complex cases (eg, insomnia, schizophrenia management), the quality of ChatGPT’s outputs declined, with some recommendations deemed inappropriate or potentially harmful [39]. When benchmarked against mental health professionals, ChatGPT demonstrated superior adherence to clinical guidelines in the management of depression [50] and comparable performance in deprescribing benzodiazepines [38]. Moreover, ChatGPT tended to suggest a broader range of proactive treatments (eg, general practitioner, counselor, psychiatrist, CBT, and lifestyle changes), while mental health professionals leaned more on targeted interventions such as psychiatric consultation and specific medication [15,52].

In terms of model version, GPT-4 generally showed the best performance, generating plausible, evidence-based interventions [37,38]. Still, it can generate ambiguous or unsafe outputs in complex cases. GPT-3.5 performed well in some areas, such as adherence to depression treatment guidelines, but may also produce serious errors.

#### Prognosis

Of all 3 studies evaluated ChatGPT’s ability to predict mental health trajectories. Across all studies, ChatGPT consistently predicted lower recovery rates than those offered by mental health practitioners or other AI models [15,40,83]. Specifically, ChatGPT-3.5 generated more negative short-term outcome predictions, whereas ChatGPT-4 exhibited greater pessimism regarding long-term mental health outcomes [40,83].

## Discussion

### Characteristics of Applications

Since its release in November 2022, ChatGPT has sparked extensive discussions in the mental health care sector [20,85]. However, its performance in conducting various clinical tasks has received less attention. This scoping review provides an insight into the clinical applications of ChatGPT in mental health care and its current empirical evidence.

The landscape of clinical use of ChatGPT is expanding, albeit unevenly, with a focus on detection, counseling, and treatment of a wide range of mental health problems, indicating the perceived value of ChatGPT to augment psychological services, especially where access is limited. However, its relatively infrequent use in areas requiring higher clinical accountability—such as prognosis and decision-making—suggests ongoing concerns about reliability, risk, and ethical responsibility [20]. Moreover, the widespread focus on standard ChatGPT, with minimal use of customized or fine-tuned models, represents a missed opportunity to strengthen context-sensitive adaptations critical for safe and effective clinical deployment [86]. Most clinical applications of ChatGPT in mental health care are primarily designed to be used for adults’ mental problems, with far fewer tools to benefit children and adolescents. This imbalance is striking, as these younger “Digital Natives” are often the earliest adopters of new technologies, and neglecting their needs risks creating a critical gap in safe, developmentally appropriate mental health support [87]. From a methodological stance, there is an overreliance on prompt-based experiment designs, based on simulations, without involving an interaction of real-world users. Even fewer studies involved clinical populations, which raises serious questions about whether ChatGPT is ready to be deployed at a large scale in mental health care services.

### Main Findings

#### Detection

Overall, the evidence for detection is mixed to generally favorable, depending on task and comparator. One of the most compelling findings is ChatGPT’s performance in binary diagnostic classification and differential diagnosis, which is comparable to or, in most cases, surpasses the performance of mental health practitioners as well as other AI models [11,45,52,69]. Meanwhile, accuracy is limited when prompted with more specialized tasks such as estimating the severity of a mental health condition [13], assigning a psychiatric diagnosis in a highly heterogeneous clinical presentation’s data [11], or assessing the risk of suicide [12,81]. These inconsistencies suggest that, although ChatGPT might perform well in identifying generalized constellations of symptoms, it encounters significant challenges in more specialized tasks and high-risk clinical scenarios. This strength may overestimate its use in real-world clinical assessment. Mental health presentations are rarely clear-cut; most patients present with comorbidities, overlapping symptom constellations, and fluctuating courses that blur diagnostic boundaries [88,89]. In such contexts, reliance on categorical outputs risks oversimplification, misclassification, and neglect of clinically relevant nuances. Effective assessment requires dimensional evaluation, consideration of differential diagnoses, and integration of psychosocial context—tasks that extend beyond binary classification and remain challenging for ChatGPT.

#### Counseling and Treatment

When deployed for counseling and treatment purposes, the overall evidence is generally weaker, with selective strengths in psychoeducation and low-intensity support. More specifically, ChatGPT shows promise in emulating therapeutic dialogue, maintaining conversational flow, approximating empathy, using therapeutic vocabulary, and providing simple therapeutic strategies [18,19,36,41,46,55,80]. It also demonstrates good capability to perform specific structured counseling tasks such as cognitive reframing and more abstract tasks such as psychodynamic conceptualizations [17,68]. These assets make ChatGPT a reliable tool for use in early engagement, psychoeducation, structured and specific clinical tasks, or in situations where traditional care is inaccessible [90]. Moreover, ChatGPT can simulate coherent therapeutic dialogue, but it also facilitates symptom reduction when tested directly with clinical or general populations for treatment outcomes [49,54,58,68].

However, one of the most disturbing findings is that, although ChatGPT might seem able to produce plausible therapeutic information, this plausibility is often only at a surface level, since its responses consistently lack accurate references or external referrals, raising serious ethical concerns. This result is per previous research, highlighting the ChatGPT tendency towards inaccurate or fabricated referencing [91]. Additionally, ChatGPT outputs are limited by a lack of contextual awareness, personalized memory, and therapeutic depth. This is particularly problematic when dealing with complex clinical presentations or sensitive, high-risk clinical scenarios that often require more than procedural knowledge [92]. In its current standard form, while ChatGPT might be considered broadly capable, it is not yet optimized for nuanced therapeutic engagement. It may underperform in domains requiring fine-grained emotional inference or crisis-specific support.

#### Clinical Decision Facilitation

Overall, the evidence for clinical decision facilitation is generally favorable, but it depends on the complexity of the clinical case. More specifically, ChatGPT demonstrates a strong alignment with evidence-based guidelines for managing specific mental health conditions. However, like detection tasks, the recommendations made by ChatGPT become less reliable and, in some instances, even dangerous, as the complexity of clinical cases increases [14,39]. These results are consistent with research in various medical contexts, where the complexity of the clinical presentation moderates the performance of AI tools in clinical management [93].

While acknowledging its limitations in detection, counseling and treatment, as well as in clinical decision facilitation tasks, it must be noted that in studies assessing ChatGPT’s relative performance, there is a tendency to approximate or even outperform mental health practitioners, as well as other AI tools. This positions ChatGPT as a potential benchmark in AI-driven mental health care, setting a new standard for performance expectations in clinical practice.

#### Prognosis

Prognosis remains an exploratory and underdeveloped application of ChatGPT. The capabilities of ChatGPT represent an area of grave concern, given the tendency to provide an overly pessimistic prognosis for mental health problems. This type of outlook can have important implications for the clinical population, reducing hope and motivation to seek or continue mental health specialized treatment [94].

### Factors Accounting for Performance Variability

Although ChatGPT shows potential in conducting clinical tasks related to mental health care, research consistently fails to replicate the positive findings regarding performance. Besides the complexity of clinical tasks and presentations, another potential explanation for these inconsistencies might be related to the prompting and the level of pretraining used in the experimental testing [95]. Indeed, previous research has shown that the performance of ChatGPT in carrying out various tasks is highly dependent on the prompting engineering—namely, on how much task-specific information or training the model is given [96,97]. Several studies included in the current review have explicitly addressed this issue, showing, for example, that adding more examples in the prompt on how to carry out the detection tasks enhances the ChatGPT detection capabilities compared to zero-shot prompting condition, where ChatGPT relies purely on its pretrained knowledge to understand the task from the instructions users write in the prompt [10,63,74]. Similarly, use of the chain-of-thought technique improves diagnostic accuracy, since the model is encouraged to reason step-by-step—explicitly outlining its thought process—before arriving at a diagnostic or evaluative conclusion [63]. Additionally, a study showed that providing multimodal input, namely speech rhythm and rate, besides text-based data, increased ChatGPT’s accuracy in distinguishing between anxiety and depression [73]. In counseling and treatment, encouraging development is the growing evidence regarding the superiority of customized ChatGPTs, suggesting that specific domain optimization maximizes the benefits across the mental health domain, by addressing some of the limitations of generic AI models [18,36]. Another key moderator of ChatGPT’s performance in clinical practice is the model version, with newer iterations like GPT-4 generally outperforming GPT-3.5, though not consistently across all tasks. These results indicate that advances improve overall reliability but do not eliminate domain-specific weaknesses.

### Implications

The findings of this review can serve as a guide to inform clinical practice regarding which type of ChatGPT applications and under which specific conditions can or cannot be reliably, safely, and confidently used, and which cannot. ChatGPT use should be limited to simple detection tasks such as binary decisions in initial screenings, triage, and continuous monitoring—if it examines or focuses on well-defined symptom constellations. It can also be used to manage and assist with counseling and intervention for simple and straightforward tasks and for simple clinical presentations, making it suitable for psychoeducation, low-intensity psychological treatments, and for support or cases where immediate care is not available. Within university counseling centers, such applications could help manage high service demand by providing first-line psychoeducational support and triaging students. In community mental health centers, ChatGPT could serve as a scalable adjunct to extend care to underserved populations, particularly in rural or low-resource contexts. In hospital-based or specialized clinical programs, its role may be more appropriately limited to intake assistance, between-session monitoring, or delivery of standardized interventions that complement provider-led care. However, given that the existing evidence with real-world patients and multicultural populations is scarce, implementation in these types of settings needs to be done with high caution. Additionally, our review suggests that ChatGPT in clinical practice should be regarded as merely a complementary tool and not a substitute for traditional mental health care, especially in complex or high-risk situations, where the value of human judgment and experience in decision-making is irreplaceable [41]. Additionally, when possible, users should choose fine-tuned or customized ChatGPT models over generic ones, because the former provide a higher level of sophistication and specificity [86,98]. While ChatGPT could be beneficial in assisting detection, counseling, and treatment, as well as in facilitating clinical decision-making for simple case presentations, both mental health experts and the clinical population should avoid turning to ChatGPT to forecast the trajectories of having mental health disorders, given its overly pessimistic outlook.

### Limitations and Recommendations for Future Research

Several limitations of the current research must be noted. First, the inclusion of gray literature can pose issues regarding the quality of the study. However, in a fast-paced domain such as ChatGPT use, gray literature enhances comprehensiveness and timeliness of available evidence [99]. As this was a scoping review, we did not conduct a formal quality appraisal of included studies, consistent with Joanna Briggs Institute and PRISMA-ScR (Preferred Reporting Items for Systematic reviews and Meta-Analyses extension for Scoping Reviews) guidance [30,33]. While the inclusion of gray literature broadened the scope of evidence, it also introduced variability in methodological rigor. Findings should therefore be interpreted with caution and regarded as exploratory, highlighting areas where more robust, peer-reviewed research is needed. Second, the methodology used preponderantly to test the performance of ChatGPT, namely prompt experiments, limits the conclusions regarding the ecological validity and how service users interpret or respond to AI outputs. Therefore, more rigorous testing designs are needed, including randomized controlled trials, exploring the additional benefits of using ChatGPT in traditional mental health care. Third, studies, including real-world users, are subject to demographic and self-selection biases, as they involve mostly young, highly educated adults who are likely to be more technologically literate and more open to digital tools, limiting generalizability.

Fourth, an important limitation emerges from the metrics used to assess ChatGPT’s performance. Accuracy or quality of answers to queries, as well as sophistication of conversation, do not equate with clinical efficacy and do not capture the process and mechanisms underlying its use, which are the main criteria for evidence-based practice in mental health care [100]. Therefore, future research should move beyond these metrics to assess whether ChatGPT use leads to symptom reduction and how it works. On the other hand, it cannot be asserted with certainty that the negative findings related to ChatGPT performance reflect actual AI deficits or that they are an artifact of distrust, negative perception, and attitudes of those who conducted the performance assessment. Algorithm aversion is a well-documented phenomenon in the AI field, referring to a default skepticism, a cognitive bias, where individuals distrust algorithm decisions and recommendations [101]. In mental health care, this aversion can lead practitioners and patients to favor human judgment over AI, even when AI demonstrates superior performance. For example, it has been shown that general trust in ChatGPT was a significant predictor of its perceived usefulness in clinical practice among health care practitioners [102]. Moreover, even the mere belief in AI involvement can diminish patients’ trust in medical and mental health-related advice, despite it being identical to that provided by human experts [103,104]. Addressing the main concerns related to trust, privacy, and ethics through education, transparent evaluation frameworks, and involving mental health care professionals in the development process is crucial for successfully adopting ChatGPT in mental health settings. Another significant issue in the use of ChatGPT for clinical applications in mental health care is related to the outdated training data it relies on. Most of the studies included tested ChatGPT 3.5 and 4, for which the cut-off date of training is September 2023; consequently, the clinical application does not integrate the latest developments. This aspect might be especially problematic in the mental health care domain, where clinical protocols for mental health disorder management are subject to ongoing updates, informed by new research findings [105].

Future research integrating ChatGPT in mental health clinical practice would also benefit from a multidisciplinary and coparticipatory approach. For example, given the encouraging results of fine-tuned and customized ChatGPT models, a further step would be an ongoing collaboration between AI and mental health experts in developing appropriate prompts for end users. Participatory methods provide one means of ensuring that AI-based solutions for mental health care are designed to meet users’ needs and therefore promote longer-term engagement [106]. The broader implications of deploying ChatGPT in mental health contexts must be addressed. The deployment of ChatGPT must be done within the existing and evolving regulatory and ethical frameworks [107]. A responsible integration of ChatGPT in mental health care involves built-in safeguarding mechanisms for accurate referrals, real-time escalation protocols for critical situations, and transparent accountability structures [107].

Future developments for ChatGPT in mental health care should prioritize training on domain-specific datasets (eg, psychiatric case notes, suicide risk assessments, and culturally diverse dialogues), and integration with evidence-based frameworks to enhance accuracy and therapeutic relevance [108]. Embedding established guidelines (*Diagnostic and Statistical Manual of Mental Disorders*, fifth edition, National Institute for Health and Care Excellence, and American Psychological Association recommendations) into model prompts or training and structured approaches such as CBT or acceptance and commitment therapy could make output more clinically reliable and standardized. Prognostic accuracy also requires improvement, through calibration with longitudinal clinical data, which could reduce the current negative bias [109]. Furthermore, enhancing cultural and contextual sensitivity through diverse training datasets would make the technology more equitable across populations [110].

In conclusion, this scoping review highlights the dual promise and perils of integrating ChatGPT into mental health care. While its scalability, immediacy, and overall diagnostic accuracy in categorical decisions and good therapeutic abilities make it a good candidate for addressing the need for immediate care, especially where the human workforce is not available, several limitations emphasize the need for cautious deployment in real life and clinical practice. The pitfalls include underperformance in complex and high-risk clinical situations, outputs lacking nuanced clinical reasoning and reliable references, and raising ethical and safety concerns. Consequently, at this moment, ChatGPT should be integrated as a supportive, not standalone, tool in mental health care, with careful oversight and adherence to ethical frameworks to ensure safety and effectiveness. Finally, we consider it crucial to address not only the inherent limitations of ChatGPT itself but also the general perception of users, particularly mental health practitioners, regarding the deployment of this tool in clinical practice. The default skepticism of users might contribute to the dismissal of this tool, ignoring its tremendous potential.

## Supplementary material

10.2196/81204 Multimedia Appendix 1 Search string sample.

10.2196/81204 Multimedia Appendix 2 Categories, components, and definitions used for data extraction and categorization.

10.2196/81204 Multimedia Appendix 3 Characteristics of the included studies.

10.2196/81204 Multimedia Appendix 4 Main findings on ChatGPT performance.

10.2196/81204 Checklist 1 PRISMA-ScR (Preferred Reporting Items for Systematic reviews and Meta-Analyses extension for Scoping Reviews) Checklist.

## Abbreviations

AI: artificial intelligence
CBT: cognitive behavioral therapy
LLM: large language model
PRISMA: Preferred Reporting Items for Systematic Reviews and Meta-Analyses
PRISMA-ScR: Preferred Reporting Items for Systematic reviews and Meta-Analyses extension for Scoping Reviews
