# Supplementary material for: ChatGPT Clinical Use in Mental Health Care: Scoping Review of Empirical Evidence
Source: JMIR Ment Health. 2025 Dec 24;12:e81204. doi: 10.2196/81204 (PMC12735656; doi:10.2196/81204)
Supplement: Multimedia Appendix 1 [file mental-v12-e81204-s001.docx]

**Multimedia Appendix 1.** Search String Sample

**PubMed**

(ChatGPT[Title/Abstract] OR GPT[Title/Abstract]) AND ("mental health"[Title/Abstract] OR "mental well-being"[Title/Abstract] OR "mental wellbeing"[Title/Abstract] OR depress*[Title/Abstract] OR stress*[Title/Abstract] OR anxi*[Title/Abstract] OR schizophrenia[Title/Abstract] OR bipolar[Title/Abstract] OR ADHD[Title/Abstract] OR autism[Title/Abstract] OR ASD[Title/Abstract] OR PTSD[Title/Abstract] OR "eating disorder*"[Title/Abstract] OR psychology*[Title/Abstract] OR therap*[Title/Abstract] OR counsel*[Title/Abstract])
