# Supplementary material for: ChatGPT Clinical Use in Mental Health Care: Scoping Review of Empirical Evidence
Source: JMIR Ment Health. 2025 Dec 24;12:e81204. doi: 10.2196/81204 (PMC12735656; doi:10.2196/81204)
Supplement: Multimedia Appendix 2 [file mental-v12-e81204-s002.docx]

| **Multimedia Appendix 2.** Categories, components and definitions used for data extraction and categorization | |
| --- | --- |
| Category | Definition and components |
| **Bibliographical information** |  |
| Type of publication | Type of communication means used to disseminate the research to a wider audience (peer-reviewed articles, preprints, conference proceedings, theses or dissertations) |
| **Characteristics of applications** |  |
| Purpose of application | The clinical application of ChatGPT   - **Detection/assessment** – identifying early signs and symptoms of mental health condition and their severity - **Counselling and psychological treatment –** providing short- and long-term psychological support for specific mental health conditions - **Clinical decision facilitation** – supporting mental health professionals in making informed, evidence-based decisions regarding diagnosis, treatment planning, risk assessment, and patient management. - **Prognosis** – formulating expectation regarding the trajectory and outcomes of a mental health condition, based on clinical judgement, individual and contextual characteristics. |
| Mental health focus | The mental health problem that is the focus of the clinical application (e.g., depression, anxiety, general mental health) |
| Age category | Age category of intended end users of ChatGPT application (whether the application is intended for children, adolescents, or adults mental health detection, counselling and treatment, clinical decision facilitation or prognosis |
| Type of ChatGPT | Type of ChatGPT, based on the level of training   - **Standard ChatGPT** – the public, general-purpose version of ChatGPT, that operates based on default setting and knowledge - **Custom ChatGPT** – a model that allows personalization by setting specific preferences of the users; it consists in a single session instruction, without involving model retraining - **Customized ChatGPT** – Modified or fine-tuned (retrained) model to serve specific use cases, tasks, or populations. This customization can include task-specific training, domain adaptation, and integration of new data. |
| **Characteristics of research** |  |
| Study design | The design used in the study to test the ChatGPT application  **Prompt studies** – design in which researchers create input prompts, that are submitted to ChatGPT, followed by an assessment of it output; does not involve real human participants  **Uncontrolled study** – involves real human participants; the outcome is assessed pre-post, without control group  **Controlled study** – research design that involves comparison of the experimental group with a control group  **Case study** - research design that involves an in-depth, detailed examination of a specific individual, group, event, or phenomenon within its real-life context |
| Type of population (if applicable) | **General population** – consists of individuals from the broader public who are not selected based on any clinical diagnosis or mental health condition.  **Clinical population** – consists of individuals who have been diagnosed with or are currently experiencing a mental health disorder |
| Comparison element (if applicable) | The element against which ChatGPT performance in conducting clinical tasks was contrasted   - Mental health experts - Other artificial intelligence tools |
| **Main findings** |  |
| Outcomes | The performance related outcomes reported by the studies (e.g., accuracy of detection of MH, quality of responses, feasibility, usability, clinical efficacy etc.) |
| Main findings | The main results related to the performance of ChatGPT in conducting clinical tasks in mental healthcare; when available, the performance compared to MH experts or other AI tools |
