# Supplementary material for: ChatGPT Clinical Use in Mental Health Care: Scoping Review of Empirical Evidence
Source: JMIR Ment Health. 2025 Dec 24;12:e81204. doi: 10.2196/81204 (PMC12735656; doi:10.2196/81204)
Supplement: Multimedia Appendix 3 [file mental-v12-e81204-s003.docx]

| **Multimedia Appendix 3.** Characteristics of the included studies | | | | | | | | |
| --- | --- | --- | --- | --- | --- | --- | --- | --- |
| **Study** | **Type of publication** | **Purpose of application** | **Category age end users** | **MH focus** | **ChatGPT model** | **Study design** | **Participants**  **characteristics** | **Comparison**  **Element** |
| Alanezi, 2024 [1] | Peer reviewed | Counseling and treatment | Adults | General MH | Standard | Uncontrolled trial | 24 adults; clinical population; 50% 18-30 years; 75% high educational level; drop-out 0% | - |
| Alanzi et al., 2024 [2] | Peer reviewed | Counseling and treatment | Adults | Anxiety | Standard | Uncontrolled trial | 399 adults; clinical population; 41% 18-30 years; 47% high education level: drop-out 1.7% | - |
| Aleem et al., 2024 [3] | Conference | Counseling and treatment | Adults | General MH | Standard | Prompt study | - | - |
| Andrade-Arenas & Yactayo-Arias 2024 [4] | Conference | Counseling and treatment | Adults | General MH | Custom GPT | Uncontrolled trial | 15 adults; general population | - |
| Aragón et al. 2024 [5] | Conference | Detection | Adults | Depression | Standard | Prompt study | - | MH experts, BertSQUAD, T5 |
| Arbanas, 2024 [6] | Peer reviewed | Counseling and treatment | Adults | General MH | Standard | Uncontrolled trial | 89 adults; clinical population; mean age 40.1; 47% completed college; drop-out 0% | Psychiatrist and pharmacologists |
| Arcan et al., 2023 [7] | Preprint | Detection | Adults | Depression  Anxiety | Standard | Prompt study | - | XGboost, Llama-2, Distil-Roberta, Xnet, BERT, DistilBERT |
| Bartal et al., 2024 [8] | Peer reviewed | Detection | Adults | PTSD | Standard + Custom instruction | Prompt study | - | - |
| Berrezueta-Guzman et al. 2024^a^ [9] | Peer reviewed | Counseling and treatment | Children | ADHD | Custom GPT | Prompt study | - | Claude 3 |
| Berrezueta-Guzman et al., 2024^b^ [10] | Conference | Counseling and treatment | Adults | ADHD | Standard + Custom GPT | Prompt study | - | - |
| Blyler & Seligman, 2024 [11] | Peer reviewed | Clinical decision facilitation | Adults | General MH | Standard | Prompt study | - | - |
| Bužančić et al., 2024 [12] | Peer reviewed | Clinical decision facilitation | Adults | General MH | Standard | Prompt study | - | MH experts |
| Cardamone et al., 2025 [13] | Peer reviewed | Detection | Mixed –adolescents ≥10 years old and adults | General MH | Standard | Prompt study | - | MH experts |
| Danner et al., 2023 [14] | Conference | Detection | Adults | Depression | Standard | Prompt study | - | BERT |
| Dergaa et al., 2024 [15] | Peer reviewed | Clinical decision facilitation | Adults | Insomnia | Standard | Prompt study | - | - |
| Elyoseph et al., 2024 [16] | Peer reviewed | Prognosis | Adults | Depression | Standard | Prompt study | - | MH expert, BARD, Claude |
| Elyoseph & Levkovich 2023 [17] | Peer reviewed | Detection | Adults | Suicide | Standard | Prompt study | - | MH experts |
| Elyoseph & Levkovich 2024 [18] | Peer reviewed | Prognosis | Adults | Schizophrenia | Standard | Prompt study | - | MH experts, BARD, Claude |
| Eshghie & Eshghie, 2023 [19] | Preprint | Counseling and treatment | Adults | General MH | Standard | Prompt study | - | - |
| Farhat, 2024 [20] | Peer reviewed | Counseling and treatment | Adults | Depression  Anxiety | Standard | Prompt study | - | - |
| Galido et al., 2023 [21] | Peer reviewed | Detection, Clinical decision facilitation | Adults | Schizophrenia | Standard | Study case | 1 adult; clinical population; age 22 years | - |
| Ghanadian et al., 2023 [22] | Conference | Detection | Adults | Suicide | Standard + Custom instruction | Prompt study | - | BERT, DistilBERT |
| Giorgi et al., 2024 [23] | Peer reviewed | Counseling and treatment | Adults | Substance use disorder | Standard | Prompt study | - | Llama-2 |
| Giray, 2025 [24] | Peer reviewed | Counseling and treatment | Adults | General MH | Standard | Study case | 7 adults; general population | - |
| Haj et al., 2024 [25] | Peer reviewed | Detection | Adults | Schizophrenia | Standard | Prompt study | - | MH experts |
| He et al., 2024 [26] | Peer reviewed | Counseling and treatment | Adults | ASD | Standard | Prompt study | - | MH express, tailored chatbot BERNIE |
| Heston, 2023 [27] | Peer reviewed | Counseling and treatment | Adults | Depression  Suicide | Custom GPTs | Prompt study | - | - |
| Hodson & Williamson 2024 [28] | Peer reviewed | Counseling and treatment | Adults | General MH | Standard | Prompt study | - | BARD |
| Hwang et al., 2024 [29] | Peer reviewed | Counseling and treatment | Adults | General MH | Standard | Prompt study | - | - |
| Kim et al., 2024 [30] | Peer reviewed | Detection | Adults | OCD | Standard | Prompt study | - | Llama-3, Gemini PRO |
| Kishimoto et al., 2025 [31] | Peer reviewed | Counseling and treatment | Adults | Anxiety | Custom GPT | Controlled trial | 98 adults; general population; 75% 18-29 years; drop-out 8.2% | Online intervention, neutral writing task |
| Lamichhane, 2023 [32] | Preprint | Detection | Adults | Depression  Suicide | Standard | Prompt study | - | - |
| Levkovich & Elyoseph, 2023^a^ [33] | Peer reviewed | Clinical decision facilitation | Adults | Depression | Standard | Prompt study | - | Primary care physicians |
| Levkovich, 2025 [34] | Peer reviewed | Detection, Clinical decision facilitation, Prognosis | Adults | Depression  Suicide  Social phobia  Schizophrenia  PTSD | Standard | Prompt study | - | MH experts, Claude, Gemini |
| Levkovich & Elyoseph 2023^b^ [35] | Peer reviewed | Detection | Adults | Suicide | Standard | Prompt study | - | MH experts |
| Levkovich et al., 2024 [36] | Peer reviewed | Detection, Clinical decision facilitation | Children | Anxiety | Standard | Prompt study | - | MH experts, Claude, Gemini |
| Levkovich et al., 2024 [37] | Preprint | Detection | Adults | Suicide | Standard | Prompt study | - | - |
| Li et al., 2024 [38] | Peer reviewed | Detection | Adults | General MH | Standard | Prompt study | - | Llama-2, BARD |
| Manole et al., 2024 [39] | Peer reviewed | Counseling and treatment | Adults | Anxiety | Custom GPT | Uncontrolled trial | 50 adults; clinical population; mean age 26.3; 73% high educational level; drop-out – 0% | - |
| Maurya et al., 2025 [40] | Peer reviewed | Counseling and treatment | Adults | General MH | Standard | Prompt study | - | - |
| McBain et al., 2025 [41] | Peer reviewed | Counseling and treatment | Adults | Suicide | Standard | Prompt study | - | MH experts, Claude Sonnet, Gemini |
| McFayden et al., 2024 [42] | Peer reviewed | Counseling and treatment | Adults | ASD | Standard | Prompt study | - | - |
| Melo et al., 2024 [43] | Peer reviewed | Counseling and treatment | Adults | General MH | Standard | Controlled trial | 12 adults; clinical population; mean age ChatGPT group – 27 years vs. MH led intervention – 47; | MH expert led intervention (standard care) |
| Naher, 2024 [44] | Peer reviewed | Counseling and treatment | Adults | General MH | Standard | Prompt study | - | MH led therapy sessions conversations transcripts |
| Nedilko, 2023 [45] | Conference | Detection | Adults | Depression | Standard + Custom instruction | Prompt study | - | - |
| Park et al., 2023 [46] | Conference | Counseling and treatment | Adults | General MH | Standard | Prompt study | - | MUSE ALPHA chatbot |
| Parker & Spoelma, 2023 [47] | Peer reviewed | Counseling and treatment | Adults | Bipolar disorder | Standard | Prompt study | - | - |
| Russel et al., 2024 [48] | Peer reviewed | Counseling and treatment | Adults | Substance use disorder | Standard | Prompt study | - | - |
| Sezgin et al., 2023 [49] | Peer reviewed | Counseling and treatment | Adults | Depression | Standard | Prompt study | - | BARD, Google |
| Shin et al., 2024 [50] | Peer reviewed | Detection | Adults | Depression | Standard + Custom instruction | Prompt study | - | - |
| Shinan-Altman 2024^a^ [51] | Peer reviewed | Detection | Adults | Suicide | Standard | Prompt study | - | - |
| Shinan Altman 2024^b^ [52] | Peer reviewed | Detection | Adults | Suicide | Standard | Prompt study | - | - |
| Soun & Nair, 2023 [53] | Conference | Detection | Adults | Suicide | Standard | Prompt study | - | MLP (Multilayer Perceptron), LSTM (Long Short-Term Memory), T-LSTM (Time-aware LSTM), FAST, GPols, BERT Base |
| Spallek et al., 2023 [54] | Peer reviewed | Counseling and treatment | Adults | Substance use disorder | Standard | Prompt study | - | MH experts |
| Spitale et al., 2024 [55] | Preprint | Detection | Adults | Depression | Standard | Prompt study | - | BARD, Llama-2 |
| Tao et al., 2023 [56] | Conference | Detection | Adults | Anxiety  Depression | Standard | Prompt study | - | - |
| Van Meter et al., 2025 [57] | Peer reviewed | Counseling and treatment | Adults | Suicide | Standard | Prompt study | - | Bing Copilot, Gemini |
| Wang & Li, 2024 [58] | Peer reviewed | Counseling and treatment | Adults | Depression | Standard | Controlled trial | 15 elderly; general population; mean age – 79 years: drop-out – 73% | MH experts led mindfulness intervention |
| Wei et al., 2023 [59] | Peer reviewed | Detection | Children and adolescents | Neurodevelopmental disorders | Standard | Prompt study | - | Pediatricians |
| Woodnutt et al., 2024 [60] | Peer reviewed | Clinical decision facilitation | Adults | Self-harm | Standard | Prompt study | - | - |
|  |  |  |  |  |  |  |  |  |

**References**

1.  Alanzi TM, Alharthi A, Alrumman S, et al. ChatGPT as a psychotherapist for anxiety disorders: An empirical study with anxiety patients. *Nutr Health*. Published online 2024. doi:10.1177/02601060241281906

2. Alanezi F. Assessing the Effectiveness of ChatGPT in Delivering Mental Health Support: A Qualitative Study. *J Multidiscip Healthc*. 2024;17:461-471. doi:10.2147/JMDH.S447368

3. Aleem M, Zahoor I, Naseem M. Towards culturally adaptive large language models in mental health: Using ChatGPT as a case study. In: P*roceedings of the Companion Publication of the ACM CSCW Conference;* 2024. p. 240–247. doi:10.1145/3678884.3681858.

4. Andrade-Arenas L, Yactayo-Arias C. Chatbot with ChatGPT technology for mental wellbeing and emotional management. *IAES Int J Artif Intell*. 2024;13(3):2635-2644. doi:10.11591/ijai.v13.i3.pp2635-2644

5. Aragón ME, Parapar J, Losada DE. Delving into the Depths: Evaluating Depression Severity through BDI-biased Summaries. In: 2024:12-22. https://www.scopus.com/inward/record.uri?eid=2-s2.0-85189758387&partnerID=40&md5=1b42db824c8840cf9a75710f3b206e01

6. Arbanas G, Periša A, Biliškov I, Sušac J, Badurina M, Arbanas D. Patients prefer human psychiatrists over chatbots: a cross-sectional study. *Croat Med J*. 2025;66(1):13-19.

7. Arcan M, Niland DP, Delahunty F. An assessment on comprehending mental health through large language models [preprint]. arXiv. Published January 9, 2024. arXiv:2401.04592v2. doi:10.48550/arXiv.2401.04592

8. Bartal A, Jagodnik KM, Chan SJ, Dekel S. AI and narrative embeddings detect PTSD following childbirth via birth stories. *Sci Rep*. 2024;14(1). doi:10.1038/s41598-024-54242-2

9. Berrezueta-Guzman S, Kandil M, Martín-Ruiz ML, Pau de la Cruz I, Krusche S. Future of ADHD Care: Evaluating the Efficacy of ChatGPT in Therapy Enhancement. Healthc Switz. 2024;12(6). doi:10.3390/healthcare12060683

10. Berrezueta‑Guzman S, Kandil M, Martín‑Ruiz ML, Pau‑de‑la‑Cruz I, Krusche S. Exploring the efficacy of robotic assistants with ChatGPT and Claude in enhancing ADHD therapy: innovating treatment paradigms. In: *Proceedings of the 2024 International Conference on Intelligent Environments (IE)*; 2024. p. 25–32. doi:10.1109/IE61493.2024.10599903.

11. Blyler AP, Seligman MEP. AI assistance for coaches and therapists. *J Posit Psychol*. 2024;19(4):592-598. doi:10.1080/17439760.2023.2257666

12. Bužančić I, Belec D, Držaić M, et al. Clinical decision-making in benzodiazepine deprescribing by healthcare providers vs. AI-assisted approach. *Br J Clin Pharmacol*. 2024;90(3):662-674. doi:10.1111/bcp.15963

13. Cardamone NC, Olfson M, Schmutte T, et al. Classifying Unstructured Text in Electronic Health Records for Mental Health Prediction Models: Large Language Model Evaluation Study. *JMIR Med Inform*. 2025;13. doi:10.2196/65454

14. Danner M, et al. Advancing mental health diagnostics: GPT-based method for depression detection. In: *Proceedings of the 62nd Annual Conference of the Society of Instrument and Control Engineers (SICE)*; 2023. p. 1290–1296. doi:10.23919/SICE59929.2023.10354236.

15. Dergaa I, Fekih-Romdhane F, Hallit S, et al. ChatGPT is not ready yet for use in providing mental health assessment and interventions. *Front PSYCHIATRY*. 2024;14. doi:10.3389/fpsyt.2023.1277756

16. Elyoseph Z, Levkovich I, Shinan-Altman S. Assessing prognosis in depression: Comparing perspectives of AI models, mental health professionals and the general public. *Fam Med Community Health*. 2024;12(Suppl 1). doi:10.1136/fmch-2023-002583

17. Elyoseph Z, Levkovich I. Beyond human expertise: the promise and limitations of ChatGPT in suicide risk assessment. *Front PSYCHIATRY*. 2023;14. doi:10.3389/fpsyt.2023.1213141

18. Elyoseph Z, Levkovich I. Comparing the Perspectives of Generative AI, Mental Health Experts, and the General Public on Schizophrenia Recovery: Case Vignette Study. *JMIR Ment Health*. 2024;11:e53043. doi:10.2196/53043

19. Eshghie M, Eshghie M. ChatGPT as a therapist assistant: A suitability study [preprint]. *arXiv*. Published April 19, 2023. arXiv:2304.09873v1. doi:10.48550/arXiv.2304.09873.

20. Farhat F. ChatGPT as a Complementary Mental Health Resource: A Boon or a Bane. *Ann Biomed Eng*. 2024;52(5):1111-1114. doi:10.1007/s10439-023-03326-7

21. Galido PV, Butala S, Chakerian M, Agustines D. A Case Study Demonstrating Applications of ChatGPT in the Clinical Management of Treatment-Resistant Schizophrenia. *CUREUS J Med Sci*. 2023;15(4). doi:10.7759/cureus.38166

22. Ghanadian H, Nejadgholi I, Al Osman H. ChatGPT for suicide risk assessment on social media: Quantitative evaluation of model performance, potentials and limitations. In: *Proceedings of the 13th Workshop on Computational Approaches to Subjectivity, Sentiment & Social Media Analysis (WASSA)*; 2023. p. 172–183. doi:10.18653/v1/2023.wassa-1.16.

23. Giorgi S, Isman K, Liu T, Fried Z, Sedoc J, Curtis B. Evaluating generative AI responses to real-world drug-related questions. *Psychiatry Res*. 2024;339. doi:10.1016/j.psychres.2024.116058

24. Giray L. Cases of Using ChatGPT as a Mental Health and Psychological Support Tool. *J Consum Health INTERNET*. 2025;29(1):29-48. doi:10.1080/15398285.2024.2442374

25. Haj MEL, Raffard S, Besche-Richard C. Decoding schizophrenia: ChatGPT’s role in clinical and neuropsychological assessment. *Schizophr Res*. 2024;267:84-85. doi:10.1016/j.schres.2024.03.031

26. He W, Zhang W, Jin Y, Zhou Q, Zhang H, Xia Q. Physician Versus Large Language Model Chatbot Responses to Web-Based Questions From Autistic Patients in Chinese: Cross-Sectional Comparative Analysis. *J Med Internet Res*. 2024;26. doi:10.2196/54706

27. Heston TF. Safety of Large Language Models in Addressing Depression. *CUREUS J Med Sci*. 2023;15(12). doi:10.7759/cureus.50729

28. Hodson N, Williamson S. Can Large Language Models Replace Therapists? Evaluating Performance at Simple Cognitive Behavioral Therapy Tasks. *Jmir Ai*. 2024;3:e52500. doi:10.2196/52500

29. Hwang G, Lee DY, Seol S, et al. Assessing the potential of ChatGPT for psychodynamic formulations in psychiatry: An exploratory study. *Psychiatry Res*. 2024;331. doi:10.1016/j.psychres.2023.115655

30. Kim J, Leonte KG, Chen ML, et al. Large language models outperform mental and medical health care professionals in identifying obsessive-compulsive disorder. *Npj Digit Med*. 2024;7(1). doi:10.1038/s41746-024-01181-x

31. Kishimoto T, Hao X, Chang T, Luo Z. Single online self-compassion writing intervention reduces anxiety: With the feedback of ChatGPT. *Internet Interv*. 2025;39. doi:10.1016/j.invent.2025.100810

32. Lamichhane B. Evaluation of ChatGPT for NLP‑based mental health applications [preprint]. arXiv. Published March 28, 2023. arXiv:2303.15727. doi:10.48550/arXiv.2303.15727

33. Levkovich I, Elyoseph Z. Identifying depression and its determinants upon initiating treatment: ChatGPT versus primary care physicians. *Fam Med Community Health*. 2023;11(4). doi:10.1136/fmch-2023-002391

34. Levkovich I. Evaluating Diagnostic Accuracy and Treatment Efficacy in Mental Health: A Comparative Analysis of Large Language Model Tools and Mental Health Professionals. *Eur J Investig Health Psychol Educ*. 2025;15(1). doi:10.3390/ejihpe15010009

35. Levkovich I, Elyoseph Z. Suicide risk assessments through the eyes of ChatGPT-3.5 versus ChatGPT-4: Vignette study. *JMIR Ment Health*. 2023;10. doi:10.2196/51232

36. Levkovich I, Rabin E, Brann M, Elyoseph Z. Large language models outperform general practitioners in identifying complex cases of childhood anxiety. *Digit Health*. 2024;10. doi:10.1177/20552076241294182

37. Levkovich I, Shinan-Altman S, Elyoseph Z. Can large language models be sensitive to culture suicide risk assessment? *J Cult Cogn Sci*. 2024;8(3):275-287. doi:10.1007/s41809-024-00151-9

38. Li DJ, Kao YC, Tsai SJ, et al. Comparing the performance of ChatGPT GPT-4, Bard, and Llama-2 in the Taiwan Psychiatric Licensing Examination and in differential diagnosis with multi-center psychiatrists. *Psychiatry Clin Neurosci*. 2024;78(6):347-352. doi:10.1111/pcn.13656

39. Manole A, Cârciumaru R, Brînzaș R, Manole F. Harnessing AI in Anxiety Management: A Chatbot-Based Intervention for Personalized Mental Health Support. *Inf Switz*. 2024;15(12). doi:10.3390/info15120768

40. Maurya RK, Montesinos S, Bogomaz M, DeDiego AC. Assessing the use of ChatGPT as a psychoeducational tool for mental health practice. *Couns Psychother Res*. 2025;25(1). doi:10.1002/capr.12759

41. McBain RK, Cantor JH, Zhang LA, et al. Competency of Large Language Models in Evaluating Appropriate Responses to Suicidal Ideation: Comparative Study. *J Med Internet Res*. 2025;27:e67891. doi:10.2196/67891

42. McFayden TC, Bristol S, Putnam O, Harrop C. ChatGPT: Artificial Intelligence as a Potential Tool for Parents Seeking Information About Autism. *Cyberpsychology Behav Soc Netw*. 2024;27(2):135-148. doi:10.1089/cyber.2023.0202

43. Melo A, Silva I, Lopes J. ChatGPT: A Pilot Study on a Promising Tool for Mental Health Support in Psychiatric Inpatient Care. *Int J Psychiatr Trainees*. 2024;2(2). doi:10.55922/001c.92367

44. Naher J. Can ChatGPT provide a better support: a comparative analysis of ChatGPT and dataset responses in mental health dialogues. *Curr Psychol*. 2024;43(28):23837-23845. doi:10.1007/s12144-024-06140-z

45. Nedilko A. Team Bias Busters@LT-EDI: Detecting signs of depression with generative pretrained transformers. In: *Proceedings of the Third Workshop on Language Technology for Equality, Diversity, and Inclusion (LT-EDI), in Recent Advances in Natural Language Processing (RANLP);* September 7, 2023; Varna, Bulgaria. p. 138-143. doi:10.26615/978-954-452-084-7_020.

46. Park H, Jung MR, Ji M, Kim J, Oh U. Muse Alpha: Primary study of AI chatbot for psychotherapy with Socratic methods. In: *Proceedings of the 2023 Congress in Computer Science, Computer Engineering, & Applied Computing (CSCE)*; 2023; Las Vegas, NV, USA. p. 2692-2693. doi:10.1109/CSCE60160.2023.00431. doi:10.1109/CSCE60160.2023.00431

47. Parker G, Spoelma MJ. A chat about bipolar disorder. *Bipolar Disord*. 2024;26(3):249-254. doi:10.1111/bdi.13379

48. Russell AM, Acuff SF, Kelly JF, Allem JP, Bergman BG. ChatGPT-4: Alcohol use disorder responses. *Addiction*. 2024;119(12):2205-2210. doi:10.1111/add.16650

49. Sezgin E, Chekeni F, Lee J, Keim S. Clinical Accuracy of Large Language Models and Google Search Responses to Postpartum Depression Questions: Cross-Sectional Study. *J Med Internet Res*. 2023;25:e49240. doi:10.2196/49240

50. Shin D, Kim H, Lee S, Cho Y, Jung W. Using Large Language Models to Detect Depression From User-Generated Diary Text Data as a Novel Approach in Digital Mental Health Screening: Instrument Validation Study. *J Med Internet Res*. 2024;26. doi:10.2196/54617

51. Shinan-Altman S, Elyoseph Z, Levkovich I. Integrating Previous Suicide Attempts, Gender, and Age Into Suicide Risk Assessment Using Advanced Artificial Intelligence Models. *J Clin Psychiatry*. 2024;85(4). doi:10.4088/JCP.24m15365

52. Shinan-Altman S, Elyoseph Z, Levkovich I. The impact of history of depression and access to weapons on suicide risk assessment: a comparison of ChatGPT-3.5 and ChatGPT-4. *PeerJ*. 2024;12. doi:10.7717/peerj.17468

53. Soun RS, Nair A. ChatGPT for mental health applications: A study on biases. In: *Proceedings of the Third International Conference on AI‑ML Systems*; 2023. p. 1–5. doi:10.1145/3639856.36398

54. Spallek S, Birrell L, Kershaw S, Devine EK, Thornton L. Can we use ChatGPT for Mental Health and Substance Use Education? Examining Its Quality and Potential Harms. *JMIR Med Educ*. 2023;9. doi:10.2196/51243

55. Spitale M, Cheong J, Gunes H. Underneath the numbers: Quantitative and qualitative gender fairness in LLMs for depression prediction [preprint]. *arXiv*. Published June 12, 2024. arXiv:2406.08183. doi:10.48550/ARXIV.2406.08183

56. Tao Y, Yang M, Shen H, Yang Z, Weng Z, Hu B. Classifying anxiety and depression through LLMs virtual interactions: A case study with ChatGPT. In: *Proceedings of the 2023 IEEE International Conference on Bioinformatics and Biomedicine (BIBM)*; December 2023. p. 2259–2264. doi:10.1109/BIBM58861.2023.10385305.

57. Van Meter AR, Wheaton MG, Cosgrove VE, Andreadis K, Robertson RE. The Goldilocks Zone: Finding the right balance of user and institutional risk for suiciderelated generative AI queries. *PLOS Digit Health*. 2025;4(1). doi:10.1371/journal.pdig.0000711

58. Wang Y, Li S. Tech vs. Tradition: ChatGPT and Mindfulness in Enhancing Older Adults’ Emotional Health. *Behav Sci*. 2024;14(10). doi:10.3390/bs14100923

59. Wei Q, Cui Y, Wei B, Cheng Q, Xu X. Evaluating the performance of ChatGPT in differential diagnosis of neurodevelopmental disorders: A pediatricians-machine comparison. *Psychiatry Res*. 2023;327:1-3. doi:10.1016/j.psychres.2023.115351

60. Woodnutt S, Allen C, Snowden J, et al. Could artificial intelligence write mental health nursing care plans? *J Psychiatr Ment Health Nurs*. 2024;31(1):79-86. doi:10.1111/jpm.12965
