# Supplementary material for: ChatGPT Clinical Use in Mental Health Care: Scoping Review of Empirical Evidence
Source: JMIR Ment Health. 2025 Dec 24;12:e81204. doi: 10.2196/81204 (PMC12735656; doi:10.2196/81204)
Supplement: Multimedia Appendix 4 [file mental-v12-e81204-s004.docx]

| **Multimedia Appendix 4.** Main findings on ChatGPT performance | | | | | |
| --- | --- | --- | --- | --- | --- |
| **Study/first author** | **Study aim** | | **ChatGPT**  **model** | **Outcomes** | **Main findings** |
|  | | **Detection** | | | |
| Aragón et al. 2024 [1] | To evaluate the effectiveness of ChatGPT of estimating the severity of depression from a thread of users’ posts | | NR | Agreement between experts and ChatGPT on estimates of severity | - Low agreement between MH expert and ChatGPT. - ChatGPT underperformed BERTsquad and T5 in severity estimation. |
| Arcan et al., 2023 [2] | To evaluate the performance of various large language models (LLMs) in symptoms scoring from anxiety and depression questionnaires and comments | | NR | Accuracy in binary diagnosis classification | - Low absolute accuracy (F1 0.33 – 0.44) - Compared to classical machine learning (XGBoost), transformer-based models (e.g., BERT, DistilBERT, XLNet), and the LLaMA-2 model, ChatGPT showed moderate performance but was outperformed by fine-tuned transformer models, especially Distil-RoBERTa. - Accuracy varies with the variations in prompts |
| Bartal et al., 2024 [3] | To evaluate the effectiveness of ChatGPT  and the text-embedding-ada-002 (ADA) model in detecting post-traumatic stress disorder following childbirth (CB-PTSD) from childbirth narratives | | GPT-3.5 | Accuracy in binary diagnosis classification | - Low accuracy for pretrained, standard ChatGPT-3.5 models in detecting PTSD (F1 0.33) - High accuracy for fine-tuned ChatGPT (F1=0.88) |
| Cardamone et al., 2025 [4] | To compare the classification decisions in diagnosis of MH problems, made by clinical experts with those generated by a state-of-the-art LLM | | NR | Agreement between MH experts ChatGPT classification | - High agreement between MH experts and ChatGPT in distinguishing mental health problems from physical health. - Low agreement for classification of MH problems into specific categories |
| Danner et al., 2023 [5] | To test a novel artificial intelligence (AI) application for depression detection, using advanced transformer networks to analyze clinical interviews. | | GPT-3.5 and 4 | Accuracy in binary diagnosis classification | - ChatGPT 3.5 (F1=0.78) outperforms both BERT (F1=0.64) and ChatGPT 4 (F1=0.61) in depression detection. |
| Elyoseph & Levkovich, 2023 [6] | To compare ChatGPT’s assessments of  mental health indicators to those of mental health professionals in a hypothetical  case study focusing on suicide risk assessment. | | GPT-3.5 | Accuracy in risk estimation for suicide | - ChatGPT-3.5 underperformed MH experts, by underestimating the risk of suicide ideation, suicide attempts and resilience |
| Galido et al., 2023 [7] | To evaluate ChatGPT ability to correctly identify the disorder in a study case of a patient with treatment resistant schizophrenia | | NR | Ability to identify the patient’s MH disorder | - ChatGPT correctly diagnosed the patient with treatment resistant schizophrenia |
| Ghanadian et al., 2023 [8] | To examine ChatGPT’s performance  in evaluating the level of suicidality in Reddit posts | | NR | Accuracy in risk estimation for suicide | - Good accuracy of ChatGPT to detect suicidality level (F1=0.70 for few shots and 0.73 for one shot model) - However, comparing models - Albert (F1=0.74) and DistilBERT (F1=0.86) - had better accuracy than ChatGPT |
| Haj et al., 2024 [9] | To investigate whether ChatGPT can assist in neuropsychological assessment of schizophrenia. | | NR | Accuracy in binary diagnosis classification | - Comparable accuracy to MH experts in diagnosing schizophrenia from neuropsychological assessment. - When tasked with determining whether the diagnosis was made by artificial intelligence or by a fellow neuropsychologist, a second neuropsychologist responded that they cannot see the difference. |
| Kim et al., 2024 [10] | To examine the diagnostic accuracy of LLM compared to clinicians and other mental health professionals using clinical vignettes of OCD. | | GPT-4 | Accuracy in binary diagnosis classification | - Excellent accuracy for ChatGPT-4 (F1=1.00) - Better performance than Gemini PRO (F1=0.93) and Llama – (F1=0.94) |
| Lamichhane, 2023 [11] | To evaluate ChatGPT’s zero-shot classification performance in depression and suicidality detection tasks, based on user’s social media posts. | | GPT-3.5 Turbo | Accuracy in binary diagnosis classification | - High accuracy in depression detection for ChatGPT 3.5 Turbo (F1=0.86) - Low accuracy in suicidality detection (F1=0.37) |
| Levkovich 2025 [12] | To compare correct diagnosis rates across different LLM tools and mental health  professionals. | | GPT-3.5 and 4 | Accuracy in binary diagnosis classification | - ChatGPT-4 outperformed professionals and achieved perfect accuracy in detecting depression, suicidal ideation, social phobia, and PTSD (F1=1.00) but underperformed in schizophrenia cases (F1=0.55) - ChatGPT-3.5 had more consistent performance, including in schizophrenia (F1= 0.95), but severely underperformed in the suicidal ideation vignette (F1=0.05) |
| Levkovich & Elyoseph 2023^b^ [13] | To evaluate ChatGPT’s ability to assess suicide risk | | GPT-3.5 and 4 | Agreement between MH experts and ChatGPT in suicide risk estimation | - ChatGPT-4 matched expert performance on suicide attempt risk but tended to overestimate ideation and psychache, and underestimate resilience. - ChatGPT-3.5 offered estimates closer to expert norms for suicidal ideation and psychache, but underestimated suicide risk. |
| Levkovich & Elyoseph, 2024 [14] | To evaluate the ability of several LLMs (ChatGPT-3.5 and ChatGPT-4, Claude.AI, Gemini) to identify cases childhood anxiety disorder, compared with reports of GPs and MH experts | | GPT-3.5 and 4 | Recognition rate | - Better performance than GPs and MH experts in recognizing anxiety for both versions 3.5 and 4 of ChatGPT. - Lower performance than Claude.AI and Gemini |
| Levkovich et al., 2024 [15] | To investigate if ChatGPT-3.5 and ChatGPT-4 integrate cultural factors in assessing suicide risks | | GPT-3.5 and 4 | Cultural sensitivity in estimating probability of suicidal ideation, potential for suicide attempts, likelihood of severe suicide attempts, and risk of mortality from a suicidal act | - ChatGPT-3.5 is sensitive to cross-cultural distinctions when predicting suicidal risk, in all metrics. - In contrast, in the ChatGPT-4 evaluation only the likelihood of a serious suicide attempt and the risk of dying from attempted suicide was sensitive to cultural distinctions |
| Li et al., 2024 [16] | To compare the scores of ChatGPT 4, Bard and Llama-2 with those of experienced psychiatrists in conducting psychiatric differential diagnosis | | GPT-4 | Mean performance in differential diagnosis | - ChatGPT-4 outperformed BARD (M=3) and Llama-2 (M=1) in differential diagnosis accuracy - ChatGPT (M=5) had lower performance compared to MH experts (M=6.1) |
| Nedilko, 2023 [17] | To assess ChatGPT’s performance in identifying depression from social media text | | NR | Accuracy in diagnosis severity | - Zero-shot ChatGPT had poor performance in identifying severe depression (F1=0.25) - Few-shot ChatGPT improved results (F=0.53) - Fine-tuned GPT significantly outperformed both ChatGPT approaches (F1=0.68) |
| Shin et al., 2024 [18] | To assess the ability of a LLM to detect depression based on user-generated diary text through an emotional diary writing app | | GPT-3.5 | Accuracy in binary diagnosis classification | - GPT-3.5 (fine-tuned), especially when combined with Chain-of-Thought (CoT) prompting, demonstrated the best performance (F1=0.68), followed by GPT-3.5 fine-tuned (no CoT) (F1=0.67). The worse performance was achieved by standard ChatGPT (F1=0.4) |
| Shinan-Altman 2024^a^ [19] | To explore how variations in prompts, specifically  regarding past suicide attempts, gender, and age influence the risk assessments provided by ChatGPT-3 and ChatGPT-4. | | GPT-3.5 and 4 | Recognitions of suicide risk factors | - Both ChatGPT-3.5 and ChatGPT-4 recognized the importance of previous suicide attempts in predicting severe suicide risks and suicidal thoughts. - ChatGPT-4 also identified gender differences, associating men with a higher risk. - Both models disregarded age as a risk factor. |
| Shinan-Altman 2024^b^ [20] | To evaluate the effectiveness of ChatGPT-3.5 and  ChatGPT-4 in incorporating critical risk factors, namely history of depression and access to weapons, into suicide risk assessments. | | GPT-3.5 and 4 | Recognitions of suicide risk factors | - ChatGPT-4 outperforms ChatGPT-3.5 in suicide risk assessment, demonstrating greater sensitivity to clinical risk factors, higher and more cautious severity ratings, and ability to recognize complex interactions between risk factors |
| Soun & Nair, 2023 [21] | Evaluate ChatGPT-3.5's effectiveness in suicide risk classification and suicide risk severity classification | | GPT-3.5 | Accuracy in risk suicide severity | - ChatGPT 3.5 outperformed 7 of the 8 comparators AI-models, with high accuracy in both classification tasks (Suicide risk – F1=0.89; risk severity F1=0.76) - Lower performance than BERT |
| Spitale et al., 2024 [22] | To investigate gender bias of LLMs in depression detection | | GPT-3.5 and 4 | Accuracy in binary diagnosis classification across gender | - ChatGPT 3.5 and 4 outperform both Bard and Llama 2 in accurately detecting depression across gender (F1=0.80) - Sensitive to gender bias |
| Tao et al., 2023 [23] | To explore ChatGPT’s potential for depression and anxiety detection | | NR | Accuracy in differential diagnosis | - Using only text data, ChatGPT shows moderate performance in distinguishing depression from anxiety, with an F1=0.7. - Including speech rhythm and rate helped ChatGPT achieve up to 0.81 F1 score, showing it can reliably differentiate anxiety and depression when prosodic cues are added. |
| Wei et al., 2023 [24] | To assess the performance of ChatGPT -4 in differential diagnosis of neurodevelopmental disorders | | GPT-4 | Accuracy in differential diagnosis | - ChatGPT-4 achieved comparable accuracy (66.7%) to pediatricians (66.7%) in differentiating common neurodevelopmental disorders in children when provided with the assessment instruments. - When provided with additional medical vignettes, the overall accuracy of pediatricians improved to 73% while ChatGPT’s accuracy declined to 53.3%. |
|  | | **Counselling and treatment** | | | |
| Alanezi, 2024 [25] | To assess the use of ChatGPT for mental health support. | | NR | Positive and negative aspects | - Positive aspects: use for psychoeducation, psychotherapeutic exercises and self-assessment and monitoring   Negative aspects: Ethical and legal considerations, limited accuracy and reliability |
| Alanzi et al., 2024 [26] | To investigate the role of ChatGPT as a psychotherapist for anxiety disorders, examining its  effectiveness, acceptability, and potential benefits among individuals with anxiety disorders. | | NR | Therapeutic abilities (comfort, helpfulness, empathy, trustworthiness, efficacy, clarity and coherence; negative aspects) | - Moderately to highly trustworthy, effective, and coherent in its anxiety therapy replies and interactions; moderate to highly comfort and helpfulness ; moderate levels of empathy. - Negative aspects: ethics and privacy |
| Aleem et al., 2024 [27] | To explore ChatGPT role as a multicultural psychotherapist | | NR | Therapeutic abilities | - Significant limitations in memory, adaptability, listening, engagement depth, and cultural sensitivity. |
| Andrade-Arenas & Yactayo-Arias 2024 [28] | To evaluate the usability of a chatbot powered by ChatGPT for mental wellbeing | | NR | Usability | - High usability - positive experience in interacting with chatbot |
| Arbanas, 2024 [29] | To rate the level of patients’ satisfaction with responses on questions regarding mental health provided by human psychiatrists, pharmacists, and chatbot platforms | | NR | Satisfaction with mental health related responses | - Lower satisfaction with ChatGPT responses, compared to MH experts’ responses |
| Berrezueta-Guzman et al. 2024^a^ [30] | To explore the integration of ChatGPT to  improve attention deficit hyperactivity disorder (ADHD) treatments. | | NR | Therapeutic abilities | - High ratings to use engaging language, maintain interest, promote active participation, and foster a positive atmosphere in therapy sessions. - Low ratings for confidentiality and privacy |
| Berrezueta-Guzman et al. 2024^b^ [31] | To compare ChatGPT turbo and Claude Opus performance in a simulated therapy scenario to gauge their effectiveness against a clinically validated customized model. | | GPT-4 Turbo | Therapeutic abilities | - *Emotional understanding and empathy*: Customized GPT higher performance than GPT 4 turbo and Claude 3 Opus, excepting validation of patient emotions, where its performance was lower than the other 2 models. - *Communication abilities*: Customized ChatGPT outperforms the other 2 models in 4 out of 5 communication metrics. ChatGPT Turbo underperforms in all metrics, compared to both Customized GPT and Claude 3. - *Engagement and motivation*: ChatGPT turbo outperforms in encouragement of autonomy and self-expression, and sustaining patient interest, but has lower rating in positive atmosphere and motivational language, aspects where Customized GPT has the best performance - *Flexibility and adaptability*: Customized ChatGPT   demonstrates superior performance, particularly excelling in adjusting based on feedback, indicating its strong ability to learn and improve from interactions. This model also shows high adaptability to changing conversation dynamics. |
| Eshghie & Eshghie, 2023 [32] | To explore the potential of using ChatGPT as a therapist assistant to provide emotional support to individuals with mental health issues in between therapy sessions. | | NR | Therapeutic abilities | - ChatGPT can participate in positive conversations, listen attentively, and offer validation and potential coping strategies without providing explicit medical advice. |
| Farhat, 2024 [33] | To assess ChatGPT’s effectiveness in providing mental health support, particularly for issues related to anxiety and depression. | | NR | Quality of answers | - ChatGPT advises professional advice as well as effective symptom control strategies including exercise anti stress management approaches. - Despite ChatGPT repeatedly urging users to seek expert consultation, certain prompts resulted in the model generating a list of prescribed medications for the subject condition. |
| Giorgi et al., 2024 [34] | To explore the effectiveness of generative AI in answering real-world substances use and recovery questions. | | NR | Quality of answers | - While clinicians initially rated the ChatGPT-generated responses as a high quality, we discovered instances of dangerous disinformation, including disregard for suicidal ideation, incorrect emergency helplines, and endorsement of home detox. Moreover, the AI systems produced inconsistent advice depending on question phrasing. - ChatGPT underperformed compared to Llama-2 |
| Giray, 2025 [35] | To explore the emerging role of ChatGPT in mental health and psychological support. | | NR | Positive and negative aspects | - Positive aspects: Users consistently reported finding comfort, practical assistance, and immediate accessibility in these interactions. - Risks: the possibility of reliance on AI, complications in natural grieving processes, and the blurring of boundaries between AI and human connections. |
| He et al., 2024 [36] | To investigate the performance of 2 conversational agents, ERNIE Bot and ChatGPT, in supporting  individuals with ASD during web-based interactions. | | GPT-4 | Quality of answers | - ChatGPT-4 outperformed Bernie BOT in all metrics (relevance, correctness, usefulness, empathy) - Lower performance than MH experts in relevance, correctness, and usefulness |
| Heston, 2023 [37] | To assess the safety of publicly available ChatGPT-3.5 conversational agents by evaluating their responses to a patient simulation indicating worsening depression and suicidality. | | NR | Quality of answers – safety (the point to which ChatGPT makes a referral to human NH experts) | - Customized ChatGPT’s postpone referrals to a perilous extent when faced with escalating mental health risk scenarios (worsening depression and suicidal ideation) |
| Hodson & Williamson 2024 [38] | To understand whether AI could recognize an  unhelpful thought, examine its validity, and reframe it to a more helpful one. | | NR | Performance in conducting cognitive restructuring | - ChatGPT performed better than BARD at identifying cognitive biases (ChatGPT 15/20, Bard 10/20) - BARD performed superiorly at reframing unhelpful thoughts (ChatGPT 16/20, Bard 19/20), even though ChatGPT had a good performance too |
| Hwang et al., 2024 [39] | To evaluate the characteristics and appropriateness of the psychodynamic formulations created by ChatGPT | | NR | Appropriateness of psychodynamic formulations | - The psychodynamic formulations generated from a case history were rated as appropriate. |
| Kishimoto et al., 2025 [40] | To explore a convenient, single-session, and effective method for reducing anxiety using ChatGPT feedback. | | NR | Effectiveness – self-compassion and anxiety | - ChatGPT component enhances the effectiveness of the self-compassion intervention in terms of self-compassion and anxiety. |
| Manole et al., 2024 [41] | To evaluate the effectiveness of an AI-powered chatbot, developed using ChatGPT, in managing anxiety symptoms | | NR | Effectiveness – anxiety  Satisfaction with intervention | - Significant reduction in anxiety symptoms pre-post intervention - High satisfaction with the accessibility and personalization of the intervention |
| Maurya et al, 2025 [42] | To explore ChatGPT as a resource for psychoeducation in the context of mental health. | | NR | Quality of answers | - Accurate, clear and relevant responses, with an empathic tone in responses to emotionally challenging queries - It engages users by offering actionable steps and suggestions. - Consistently demonstrated ethical considerations by advising users to consult qualified professionals when necessary |
| McBain et al., 2025 [43] | To assess the competency of 3 LLMs to distinguish appropriate versus inappropriate responses when engaging individuals who exhibit suicidal ideation. | | GPT-4o | Quality of answers | - Comparable accuracy of responses with MH experts - Less accurate answers than Claude Sonet , but performed better than Gemini 1.5 |
| McFayden et al., 2024 [44] | To evaluate answers provided by ChatGPT, including basic information about autism, myths/misconceptions, and resources. | | GPT-4 | Quality of answers | - ChatGPT was largely correct, concise, and clear - It did not provide much actionable advice, which was further limited by inaccurate references and hyperlinks |
| Melo et al., 2024 [45] | To assess ChatGPT’s effectiveness as an artificial intelligence (AI) chatbot in psychiatric inpatient care. | | NR | Effectiveness – quality of life  Satisfaction with intervention | - Better efficacy for ChatGPT intervention than standard care in improving quality of life - Higher satisfaction |
| Naher, 2024 [46] | To assess ChatGPT’s performance in providing mental health support | | NR | Quality of answers | - ChatGPT outperforms a specialized dataset across all evaluated dimensions –quality of information, depth and sentiment |
| Park et al., 2023 [47] | To compare ChatGPT and Muse Alpha, a chatbot, in delivering psychotherapy-style conversations based on Socratic questioning | | NR | Performance in conduction cognitive restructuring | - Limited performance in delivering therapeutic Socratic dialogue (cognitive restructuring) |
| Parker & Spoelma, 2023 [48] | To assess the capabilities of ChatGPT in generating informative content related to bipolar disorder | | NR | Quality of responses | - ChatGPT can provide simple information about bipolar disorder and create creative content for education purposes - Lacks the ability to provide up-to-date scientific references and content. |
| Russel et al., 2024 [49] | To evaluate the quality of ChatGPT-4 responses to  AUD-related queries. | | GPT-4 | Quality of answers | - Good adherence to evidence-based practices - Few referrals to external sources |
| Sezgin et al., 2023 [50] | To assess the clinical quality of generative AI responses to questions about postpartum depression. | | GPT-4 | Quality of answers | - ChatGPT-4 outperforms BARD and Google in providing clinically relevant responses |
| Spallek et al., 2023 [51] | To explore whether ChatGPT can answer user questions and assist in developing educational health  materials for mental health and substance use | | GPT-4 | Quality of answers | - At face value, the responses seemed of good quality, but further inspection revealed substandard quality compared to material created by experts - Adherence to communication guidelines and references of evidence-based resources were poor. |
| Van Meter et al., 2025 [52] | To evaluate the quality of generative AI tool responses to suicide-related queries | | GPT-3.5 and 4 | Quality of answers | - ChatGPT 3.5 and GPT-4 were consistent in providing empathetic, safe responses, but neither included hotline numbers or much evidence-based content. - ChatGPT-3 based chatbot better performance than Bing Copilot and lower performance than Gemini |
| Wang & Li, 2024 [53] | To compare AI chatbots, such as ChatGPT 3.0, with traditional mindfulness therapies on depression in older adults. | | GPT-3 | Effectiveness - tension | - Reduction in tension in both groups but no significant difference between ChatGPT and group session mindfulness |
|  | | **Clinical decision facilitation** | | | |
| Blyler & Seligman, 2024 [54] | To explore the use of ChatGPT to see if it could recommend tailored approaches and specific interventions for coaches and therapists to use with clients | | GPT-4 | Performance in generating tailored interventions Evidence based status of the generated interventions | - ChatGPT-4 generated highly plausible coaching strategies and highly plausible specific interventions, aligned with the literature |
| Bužančić et al., 2024 [55] | To compare the clinical decision-making for benzodiazepine deprescribing between a healthcare provider and ChatGPT-4 | | GPT-4 | Agreement between experts and ChatGPT  Quality of answers | - Similar performance between ChatGPT 4 and healthcare professionals in deprescribing benzodiazepines (excellent agreement, k=95%) - GPT-4 responses were 22.1% ambiguous outputs, generic answers and inaccuracies. |
| Dergaa et al., 2024 [56] | To assess ChatGPT’s potential as a tool  for mental health professionals by  generating condition assessments and  treatment recommendations for  hypothetical patient cases presenting  with sleep issues. | | NR | Appropriateness of recommendation  Positive aspects and negative aspects | - For less complex cases, ChatGPT’s recommendations were generally appropriate. - However, with growing complexity, AI-generated medical recommendations became inappropriate and even dangerous. - Strengths: ability to provide quick responses to user queries and to simulate empathy. - Limitations: ChatGPT inability to interact with users to collect further information relevant to management of a patient’s clinical condition; inability to use critical thinking and clinical judgment to drive patient’s management. |
| Galido et al., 2023 [7] | To compare the medical management suggested by ChatGPT for a patient with treatment resistant schizophrenia to current standards of care | | NR | Ability to develop a treatment plan | - Provides evidence-based treatment recommendations, offer pharmacologic and nonpharmacologic treatments, and identify side effects of medications. |
| Levkovich & Elyoseph, 2023^a^ [57] | To compare suggested treatment protocols for mild and severe depression generated by ChatGPT with those of primary care physicians. | | GPT-3.5 | Adherence to guidelines for managing depression | - Better adherence of ChatGPT-3.5 to the accepted guidelines for managing mild and severe depression than the recommendations of primary care physicians. |
| Levkovich, 2025 [12] | To compare treatments across different LLM tools and mental health professionals. | | GPT-3.5 and 4 | Characteristics of treatment recommendations | - Both versions of ChatGPT (3.5 and 4) tended to suggest a broader range of proactive treatments, whereas professionals recommended more targeted psychiatric consultations and specific medications. - Similar performance with other AI models |
| Levkovich & Elyoseph, 2024 [14] | To compare treatment recommendations of different LLMs and general practitioners (GPs) | | GPT-3-5 and 4 | Characteristics of treatment recommendations | - Both versions of ChatGPT had a notable inclination toward recommending more intensive treatments, particularly specialized mental healthcare,   compared to GPs, who exhibited a more gradual approach |
| Woodnutt et al., 2024 [58] | To use artificial intelligence (ChatGPT) to create a mental health care plan and evaluate the quality of the output against the authors’ clinical experience and  existing guidance | | GPT-3.5 | Quality of recommendations | - ChatGPT 3.5 generated an evidence-based care plan, in line with some of the national guidance. - However, the output had significant errors, including a misattribution of substance abuse to the clinical presentation, which could lead to unmerited interventions. |
|  | | **Prognosis** | | | |
| Elyoseph et al., 2024 [59] | To compare the performance of AI models against clinicians in evaluating clinical vignettes for predicting clinical prognosis and long-term outcomes in depression. | | GPT-3.5 and 4 | The outlook of the prognosis -recovery rate | - ChatGPT 3.5 - more pessimistic short-term prognosis than MH professional - ChatGPT 4 - more pessimistic long-term prognosis |
| Elyoseph & Levkovich, 2024 [60] | To evaluate the ability of LLMs in comparison to mental health professionals to assess the prognosis of schizophrenia with and without professional treatment and the long-term positive and negative outcomes. | | GPT-3.5 and 4 | The outlook of the prognosis – recovery rate | - ChatGPT 3.5 and 4 - more pessimistic in short- and long-term prognosis than MH professional - ChatGPT 3.5 - more pessimistic short-term prognosis than Claude, BARD and GPT 4 - ChatGPT 4 - more pessimistic long-term prognosis than BARD and Claude |
| Levkovich, 2025 [12] | To compare outcomes predicted by LLM tools and mental health professionals, both for those who received help and for those who did not. | | GPT-3.5 and 4 | The outlook of the prognosis – recovery rate | - ChatGPT both versions have more pessimistic prognosis than MH experts |

**References**

1. Aragón ME, Parapar J, Losada DE. Delving into the Depths: Evaluating Depression Severity through BDI-biased Summaries. In: 2024:12-22. https://www.scopus.com/inward/record.uri?eid=2-s2.0-85189758387&partnerID=40&md5=1b42db824c8840cf9a75710f3b206e01

2. Arcan M, Niland DP, Delahunty F. An assessment on comprehending mental health through large language models [preprint]. arXiv. Published January 9, 2024. arXiv:2401.04592v2. doi:10.48550/arXiv.2401.04592

3. Bartal A, Jagodnik KM, Chan SJ, Dekel S. AI and narrative embeddings detect PTSD following childbirth via birth stories. *Sci Rep*. 2024;14(1). doi:10.1038/s41598-024-54242-2

4. Cardamone NC, Olfson M, Schmutte T, et al. Classifying Unstructured Text in Electronic Health Records for Mental Health Prediction Models: Large Language Model Evaluation Study. *JMIR Med Inform*. 2025;13. doi:10.2196/65454

5. Danner M, et al. Advancing mental health diagnostics: GPT-based method for depression detection. In: *Proceedings of the 62nd Annual Conference of the Society of Instrument and Control Engineers (SICE)*; 2023. p. 1290–1296.

6. Elyoseph Z, Levkovich I. Beyond human expertise: the promise and limitations of ChatGPT in suicide risk assessment. *Front PSYCHIATRY*. 2023;14. doi:10.3389/fpsyt.2023.1213141

7. Galido PV, Butala S, Chakerian M, Agustines D. A Case Study Demonstrating Applications of ChatGPT in the Clinical Management of Treatment-Resistant Schizophrenia. *CUREUS J Med Sci*. 2023;15(4). doi:10.7759/cureus.38166

8. Ghanadian H, Nejadgholi I, Al Osman H. ChatGPT for suicide risk assessment on social media: Quantitative evaluation of model performance, potentials and limitations. In: *Proceedings of the 13th Workshop on Computational Approaches to Subjectivity, Sentiment & Social Media Analysis (WASSA)*; 2023. p. 172–183. doi:10.18653/v1/2023.wassa-1.16.

9. Haj MEL, Raffard S, Besche-Richard C. Decoding schizophrenia: ChatGPT’s role in clinical and neuropsychological assessment. *Schizophr Res*. 2024;267:84-85. doi:10.1016/j.schres.2024.03.031

10. Kim J, Leonte KG, Chen ML, et al. Large language models outperform mental and medical health care professionals in identifying obsessive-compulsive disorder. *Npj Digit Med*. 2024;7(1). doi:10.1038/s41746-024-01181-x

11. Lamichhane B. Evaluation of ChatGPT for NLP‑based mental health applications [preprint]. arXiv. Published March 28, 2023. arXiv:2303.15727. doi:10.48550/arXiv.2303.15727

12. Levkovich I. Evaluating Diagnostic Accuracy and Treatment Efficacy in Mental Health: A Comparative Analysis of Large Language Model Tools and Mental Health Professionals. *Eur J Investig Health Psychol Educ*. 2025;15(1). doi:10.3390/ejihpe15010009

13. Levkovich I, Elyoseph Z. Suicide risk assessments through the eyes of ChatGPT-3.5 versus ChatGPT-4: Vignette study. *JMIR Ment Health*. 2023;10. doi:10.2196/51232

14. Levkovich I, Rabin E, Brann M, Elyoseph Z. Large language models outperform general practitioners in identifying complex cases of childhood anxiety. *Digit Health*. 2024;10. doi:10.1177/20552076241294182

15. Levkovich I, Shinan-Altman S, Elyoseph Z. Can large language models be sensitive to culture suicide risk assessment? *J Cult Cogn Sci*. 2024;8(3):275-287. doi:10.1007/s41809-024-00151-9

16. Li DJ, Kao YC, Tsai SJ, et al. Comparing the performance of ChatGPT GPT-4, Bard, and Llama-2 in the Taiwan Psychiatric Licensing Examination and in differential diagnosis with multi-center psychiatrists. *Psychiatry Clin Neurosci*. 2024;78(6):347-352. doi:10.1111/pcn.13656

17. Nedilko A. Team Bias Busters@LT-EDI: Detecting signs of depression with generative pretrained transformers. In: *Proceedings of the Third Workshop on Language Technology for Equality, Diversity, and Inclusion (LT-EDI), in Recent Advances in Natural Language Processing (RANLP);* September 7, 2023; Varna, Bulgaria. p. 138-143. doi:10.26615/978-954-452-084-7_020.

18. Shin D, Kim H, Lee S, Cho Y, Jung W. Using Large Language Models to Detect Depression From User-Generated Diary Text Data as a Novel Approach in Digital Mental Health Screening: Instrument Validation Study. *J Med Internet Res*. 2024;26. doi:10.2196/54617

19. Shinan-Altman S, Elyoseph Z, Levkovich I. Integrating Previous Suicide Attempts, Gender, and Age Into Suicide Risk Assessment Using Advanced Artificial Intelligence Models. *J Clin Psychiatry*. 2024;85(4). doi:10.4088/JCP.24m15365

20. Shinan-Altman S, Elyoseph Z, Levkovich I. The impact of history of depression and access to weapons on suicide risk assessment: a comparison of ChatGPT-3.5 and ChatGPT-4. *PeerJ*. 2024;12. doi:10.7717/peerj.17468

21. Soun RS, Nair A. ChatGPT for mental health applications: A study on biases. In: *Proceedings of the Third International Conference on AI‑ML Systems*; 2023. p. 1–5. doi:10.1145/3639856.36398

22. Spitale M, Cheong J, Gunes H. Underneath the numbers: Quantitative and qualitative gender fairness in LLMs for depression prediction [preprint]. *arXiv*. Published June 12, 2024. arXiv:2406.08183. doi:10.48550/ARXIV.2406.08183

23. Tao Y, Yang M, Shen H, Yang Z, Weng Z, Hu B. Classifying anxiety and depression through LLMs virtual interactions: A case study with ChatGPT. In: *Proceedings of the 2023 IEEE International Conference on Bioinformatics and Biomedicine (BIBM)*; December 2023. p. 2259–2264. doi:10.1109/BIBM58861.2023.10385305.

24. Wei Q, Cui Y, Wei B, Cheng Q, Xu X. Evaluating the performance of ChatGPT in differential diagnosis of neurodevelopmental disorders: A pediatricians-machine comparison. *Psychiatry Res*. 2023;327:1-3. doi:10.1016/j.psychres.2023.115351

25.  Alanzi TM, Alharthi A, Alrumman S, et al. ChatGPT as a psychotherapist for anxiety disorders: An empirical study with anxiety patients. *Nutr Health*. Published online 2024. doi:10.1177/02601060241281906

26. Alanezi F. Assessing the Effectiveness of ChatGPT in Delivering Mental Health Support: A Qualitative Study. *J Multidiscip Healthc*. 2024;17:461-471. doi:10.2147/JMDH.S447368

27. Aleem M, Zahoor I, Naseem M. Towards culturally adaptive large language models in mental health: Using ChatGPT as a case study. In: P*roceedings of the Companion Publication of the ACM CSCW Conference;* 2024. p. 240–247. doi:10.1145/3678884.3681858.

28. Andrade-Arenas L, Yactayo-Arias C. Chatbot with ChatGPT technology for mental wellbeing and emotional management. *IAES Int J Artif Intell*. 2024;13(3):2635-2644. doi:10.11591/ijai.v13.i3.pp2635-2644

29. Arbanas G, Periša A, Biliškov I, Sušac J, Badurina M, Arbanas D. Patients prefer human psychiatrists over chatbots: a cross-sectional study. *Croat Med J*. 2025;66(1):13-19.

30. Berrezueta-Guzman S, Kandil M, Martín-Ruiz ML, Pau de la Cruz I, Krusche S. Future of ADHD Care: Evaluating the Efficacy of ChatGPT in Therapy Enhancement. Healthc Switz. 2024;12(6). doi:10.3390/healthcare12060683

31. Berrezueta‑Guzman S, Kandil M, Martín‑Ruiz ML, Pau‑de‑la‑Cruz I, Krusche S. Exploring the efficacy of robotic assistants with ChatGPT and Claude in enhancing ADHD therapy: innovating treatment paradigms. In: *Proceedings of the 2024 International Conference on Intelligent Environments (IE)*; 2024. p. 25–32. doi:10.1109/IE61493.2024.10599903.

32. Eshghie M, Eshghie M. ChatGPT as a therapist assistant: A suitability study [preprint]. *arXiv*. Published April 19, 2023. arXiv:2304.09873v1. doi:10.48550/arXiv.2304.09873.

33. Farhat F. ChatGPT as a Complementary Mental Health Resource: A Boon or a Bane. *Ann Biomed Eng*. 2024;52(5):1111-1114. doi:10.1007/s10439-023-03326-7

34. Giorgi S, Isman K, Liu T, Fried Z, Sedoc J, Curtis B. Evaluating generative AI responses to real-world drug-related questions. *Psychiatry Res*. 2024;339. doi:10.1016/j.psychres.2024.116058

35. Giray L. Cases of Using ChatGPT as a Mental Health and Psychological Support Tool. *J Consum Health INTERNET*. 2025;29(1):29-48. doi:10.1080/15398285.2024.2442374

36. He W, Zhang W, Jin Y, Zhou Q, Zhang H, Xia Q. Physician Versus Large Language Model Chatbot Responses to Web-Based Questions From Autistic Patients in Chinese: Cross-Sectional Comparative Analysis. *J Med Internet Res*. 2024;26. doi:10.2196/54706

37. Heston TF. Safety of Large Language Models in Addressing Depression. *CUREUS J Med Sci*. 2023;15(12). doi:10.7759/cureus.50729

38. Hodson N, Williamson S. Can Large Language Models Replace Therapists? Evaluating Performance at Simple Cognitive Behavioral Therapy Tasks. *Jmir Ai*. 2024;3:e52500. doi:10.2196/52500

39. Hwang G, Lee DY, Seol S, et al. Assessing the potential of ChatGPT for psychodynamic formulations in psychiatry: An exploratory study. *Psychiatry Res*. 2024;331. doi:10.1016/j.psychres.2023.115655

40. Kishimoto T, Hao X, Chang T, Luo Z. Single online self-compassion writing intervention reduces anxiety: With the feedback of ChatGPT. *Internet Interv*. 2025;39. doi:10.1016/j.invent.2025.100810

41. Manole A, Cârciumaru R, Brînzaș R, Manole F. Harnessing AI in Anxiety Management: A Chatbot-Based Intervention for Personalized Mental Health Support. *Inf Switz*. 2024;15(12). doi:10.3390/info15120768

42. Maurya RK, Montesinos S, Bogomaz M, DeDiego AC. Assessing the use of ChatGPT as a psychoeducational tool for mental health practice. *Couns Psychother Res*. 2025;25(1). doi:10.1002/capr.12759

43. McBain RK, Cantor JH, Zhang LA, et al. Competency of Large Language Models in Evaluating Appropriate Responses to Suicidal Ideation: Comparative Study. *J Med Internet Res*. 2025;27:e67891. doi:10.2196/67891

44. McFayden TC, Bristol S, Putnam O, Harrop C. ChatGPT: Artificial Intelligence as a Potential Tool for Parents Seeking Information About Autism. *Cyberpsychology Behav Soc Netw*. 2024;27(2):135-148. doi:10.1089/cyber.2023.0202

45. Melo A, Silva I, Lopes J. ChatGPT: A Pilot Study on a Promising Tool for Mental Health Support in Psychiatric Inpatient Care. *Int J Psychiatr Trainees*. 2024;2(2). doi:10.55922/001c.92367

46. Naher J. Can ChatGPT provide a better support: a comparative analysis of ChatGPT and dataset responses in mental health dialogues. *Curr Psychol*. 2024;43(28):23837-23845. doi:10.1007/s12144-024-06140-z

47. Park H, Jung MR, Ji M, Kim J, Oh U. Muse Alpha: Primary study of AI chatbot for psychotherapy with Socratic methods. In: *Proceedings of the 2023 Congress in Computer Science, Computer Engineering, & Applied Computing (CSCE)*; 2023; Las Vegas, NV, USA. p. 2692-2693. doi:10.1109/CSCE60160.2023.00431. doi:10.1109/CSCE60160.2023.00431

48. Parker G, Spoelma MJ. A chat about bipolar disorder. *Bipolar Disord*. 2024;26(3):249-254. doi:10.1111/bdi.13379

49. Russell AM, Acuff SF, Kelly JF, Allem JP, Bergman BG. ChatGPT-4: Alcohol use disorder responses. *Addiction*. 2024;119(12):2205-2210. doi:10.1111/add.16650

50. Sezgin E, Chekeni F, Lee J, Keim S. Clinical Accuracy of Large Language Models and Google Search Responses to Postpartum Depression Questions: Cross-Sectional Study. *J Med Internet Res*. 2023;25:e49240. doi:10.2196/49240

51. Spallek S, Birrell L, Kershaw S, Devine EK, Thornton L. Can we use ChatGPT for Mental Health and Substance Use Education? Examining Its Quality and Potential Harms. *JMIR Med Educ*. 2023;9. doi:10.2196/51243

52. Van Meter AR, Wheaton MG, Cosgrove VE, Andreadis K, Robertson RE. The Goldilocks Zone: Finding the right balance of user and institutional risk for suiciderelated generative AI queries. *PLOS Digit Health*. 2025;4(1). doi:10.1371/journal.pdig.0000711

53. Wang Y, Li S. Tech vs. Tradition: ChatGPT and Mindfulness in Enhancing Older Adults’ Emotional Health. *Behav Sci*. 2024;14(10). doi:10.3390/bs14100923

54. Blyler AP, Seligman MEP. AI assistance for coaches and therapists. *J Posit Psychol*. 2024;19(4):592-598. doi:10.1080/17439760.2023.2257666

55. Bužančić I, Belec D, Držaić M, et al. Clinical decision-making in benzodiazepine deprescribing by healthcare providers vs. AI-assisted approach. *Br J Clin Pharmacol*. 2024;90(3):662-674. doi:10.1111/bcp.15963

56. Dergaa I, Fekih-Romdhane F, Hallit S, et al. ChatGPT is not ready yet for use in providing mental health assessment and interventions. *Front PSYCHIATRY*. 2024;14. doi:10.3389/fpsyt.2023.1277756

57. Levkovich I, Elyoseph Z. Identifying depression and its determinants upon initiating treatment: ChatGPT versus primary care physicians. *Fam Med Community Health*. 2023;11(4). doi:10.1136/fmch-2023-002391

58. Woodnutt S, Allen C, Snowden J, et al. Could artificial intelligence write mental health nursing care plans? *J Psychiatr Ment Health Nurs*. 2024;31(1):79-86. doi:10.1111/jpm.12965

59. Elyoseph Z, Levkovich I, Shinan-Altman S. Assessing prognosis in depression: Comparing perspectives of AI models, mental health professionals and the general public. *Fam Med Community Health*. 2024;12(Suppl 1). doi:10.1136/fmch-2023-002583

60. Elyoseph Z, Levkovich I. Comparing the Perspectives of Generative AI, Mental Health Experts, and the General Public on Schizophrenia Recovery: Case Vignette Study. *JMIR Ment Health*. 2024;11:e53043. doi:10.2196/53043
